# Characterizing the Neutrophilic Inflammation in Chronic Rhinosinusitis With Nasal Polyps

**DOI:** 10.3389/fcell.2021.793073

**Published:** 2021-12-17

**Authors:** Jian-Wen Ruan, Jie-Fang Zhao, Xue-Li Li, Bo Liao, Li Pan, Ke-Zhang Zhu, Qi-Miao Feng, Jin-Xin Liu, Zi-E Yu, Jia Song, Hai Wang, Zheng Liu

**Affiliations:** Department of Otolaryngology-Head and Neck Surgery, Tongji Hospital, Tongji Medical College, Huazhong University of Science and Technology, Wuhan, China

**Keywords:** apoptosis, chronic rhinosinusitis with nasal polyps, granulocyte colonystimulating factor, neutrophil, inflammation

## Abstract

The mechanisms underlying neutrophilic inflammation in chronic rhinosinusitis with nasal polyps (CRSwNP) remain poorly investigated. This study aimed to examine the factors that contribute to tissue neutrophilia in CRSwNP. The numbers of neutrophils and active caspase-3-positive apoptotic neutrophils in sinonasal tissues were assessed via immunofluorescence staining. The 95th percentile of tissue neutrophil numbers in control subjects was selected as a cut-off to define neutrophil-high (Neu-high) or neutrophil-low (Neu-low) nasal polyps (NPs). The levels of 34 inflammatory mediators in sinonasal tissues were analyzed using Bio-Plex assay. Purified human peripheral blood neutrophils were incubated with nasal tissue homogenates, and the apoptotic neutrophils were assessed *via* flow cytometry. The cut-off for Neu-high NPs was >10 myeloperoxidase positive cells/high-power field. Compared with Neu-low NPs, Neu-high NPs had higher tissue levels of IL-1β, IL-1Ra, IL-6, IL-8, G-CSF, MCP-1, and MIP-1α, but lower levels of IL-5, IL-13, IgE, and eosinophils. Principal component and multiple correspondence analyses revealed mixed type 1, type 2, and type 3 endotypes for Neu-low NPs, and predominant type 1 and type 3 endotypes for Neu-high NPs. Neu-high NPs had lower percentages of apoptotic neutrophils than Neu-low NPs. The numbers of neutrophils and the percentages of apoptotic neutrophils correlated with G-CSF and IL-6 levels in the NPs. Tissue homogenates from Neu-high NPs, but not those from Neu-low NPs, suppressed neutrophil apoptosis *in vitro*, which was reversed by anti-G-CSF treatment. Tissue neutrophil numbers were associated with difficult-to-treat disease in patients with CRSwNP after surgery. We propose that G-CSF promotes neutrophilic inflammation by inhibiting neutrophil apoptosis in CRSwNP.

## Introduction

Chronic rhinosinusitis with nasal polyps (CRSwNP) is a highly prevalent disorder characterized by inflammation of the sinonasal mucosa and polyp formation (Shi et al., 2015; Schleimer, 2017; Fokkens et al., 2020). It affects a considerable number of people worldwide and is responsible for substantial humanistic burden and healthcare costs (Bachert et al., 2015; Schleimer, 2017). Type 2 (T2) inflammation and tissue eosinophilia have played an important role in the pathogenesis of CRSwNP (Cao et al., 2009; Wang et al., 2016). However, the incidence of T2 and eosinophilic inflammation varies greatly across different geographic areas and populations with diverse ethnic backgrounds. Additionally, eosinophils are not the only effectors implicated in the development of CRSwNP (Cao et al., 2009; Fan et al., 2011; Wen et al., 2012; Wang et al., 2016; Zhang et al., 2017; Wang et al., 2018; Wang et al., 2019). We have previously shown enhanced infiltration of neutrophils in both eosinophilic and noneosinophilic nasal polyps (NPs) in Chinese patients (Wang et al., 2018; Cao et al., 2019). It has also been reported that NPs, which are highly eosinophilic, also have substantial levels of neutrophils in Caucasian patients (Bachert et al., 2010; Tomassen et al., 2016; Wang et al., 2016; Pothoven et al., 2017; Delemarre et al., 2021). Recent evidence has demonstrated that neutrophils may disrupt the nasal epithelial barrier and cause tissue remodeling in NPs (Shi et al., 2013; Pothoven et al., 2017). Importantly, tissue neutrophilia has been associated with a poor response to corticosteroid therapy in patients with CRSwNP (Wen et al., 2012; Liao et al., 2018). These studies underscore the important, but poorly understood role of neutrophils in the pathophysiological processes of CRSwNP. Presently, the mechanisms associated with the regulation of tissue neutrophilia in CRSwNP remain largely unknown.

Recruitment of neutrophils to the local inflammatory sites is typically controlled by neutrophil chemokines, such as chemokine (C-X-C motif) ligand (CXCL) 1, CXCL2, and CXCL8 (IL-8) (Coffelt et al., 2016; de Oliveira et al., 2016). Increased IL-8 levels in NPs have been reported in patients from both Asian and Western countries (Van Zele et al., 2006; Wang et al., 2016). Additionally, nasal epithelial cell-derived IL-36γ and Charcot-Leyden crystals, produced by eosinophils, have been found to facilitate neutrophil chemotaxis by promoting IL-8 production in NPs (Wang et al., 2018; Gevaert et al., 2020). Although these studies have improved our understanding of the mechanisms involved in the development of neutrophilic inflammation in CRSwNP, a limited number of neutrophilic inflammation biomarkers have been investigated to date. In addition to enhanced recruitment, tissue neutrophilia can also be influenced by the apoptotic properties of neutrophils in the local environment. It remains unclear whether there is a change in neutrophil survival in the setting of CRSwNP. Therefore, we aimed to perform a comprehensive and integrated analysis of neutrophilic inflammation in a large cohort of CRSwNP patients. The end goals were to characterize the immunopathological patterns associated with neutrophilic inflammation in NPs and identify the driving factors in neutrophilic inflammation in NPs.

## Materials and Methods

### Subjects and Specimens

This study was approved by the Ethics Committee of Tongji Hospital and conducted with written informed consent from every participant. Eighty-two control subjects and 375 patients with CRSwNP were enrolled. CRSwNP was diagnosed according to the current guidelines (Fokkens et al., 2020; Orlandi et al., 2021). Control subjects were those undergoing septoplasty because of anatomic variations without any other inflammatory sinonasal disorders. During the surgery, polyp, and inferior turbinate tissue samples were collected from CRSwNP patients and control subjects, respectively. The details of subjects’ characteristics are presented in Supplementary Table SE1. Of note, there was not sufficient tissue from each subject for every analysis. The sample size for each experiment is indicated in the corresponding figures and figure legends.

Among CRSwNP patients, 249 of them were involved in the clinical and immunopathological feature study. Baseline symptoms were scored by visual analog scale (VAS) method as previously described (Fokkens et al., 2020). Endoscopic findings were scored according to the Lund-Kennedy method (Fokkens et al., 2020). Computed tomography (CT) scans were graded based on the Lund-Mackay scoring system (Fokkens et al., 2020). After surgery, patients were routinely given fluticasone propionate nasal spray 200 μg twice daily, and saline irrigation twice daily. Subjects with uncontrolled symptoms were given 1–2 weeks of broad-spectrum antibiotics (usually oral cephalosporin), and/or oral prednisone tablets for 20 days (30 mg daily on days 1–5, 20 mg daily on days 6–10, 10 mg daily on days 11–15, and 5 mg daily on days 16–20) (Liao et al., 2018; Fokkens et al., 2020; Chen et al., 2021). None of patients received macrolides. Patients who did not reach an acceptable level of control (controlled or partially controlled) despite adequate surgery, intranasal corticosteroid treatment, and up to two short courses of antibiotics or systemic corticosteroids in the last year were considered to have difficult-to-treat disease (Fokkens et al., 2020).

To validate the rationality of using inferior turbinate tissue as a control in neutrophil infiltration study, normal ethmoid sinus tissues were obtained from 30 subjects receiving surgery for sinus cyst, nasal tumour or maxillofacial trauma and without rhinosinusitis or rhinitis. The neutrophil infiltration was compared between inferior turbinate and ethmoid sinus tissues. In addition, 46 healthy subjects were recruited for the peripheral blood neutrophil isolation and culture study (Supplementary Table SE2). More information is provided in the Online Supplement.

### Histology and Immunofluorescence Staining

Hematoxylin and eosin (HE) and immunofluorescence staining, and cell quantification were performed as previously described (Cao et al., 2009; Wang et al., 2018). The 95th percentile of numbers of tissue myeloperoxidase (MPO) positive neutrophils per high-power field (HPF) in control subjects was selected as a cut-off to define neutrophil-high (Neu-high) or neutrophil-low (Neu-low) NPs. More information is provided in the Online Supplement including Supplementary Table SE3 and Supplementary Table SE4.

### Measurement of Inflammatory Mediators in Nasal Tissues

Surgical tissue samples were weighed and homogenized (Liao et al., 2018). MPO and eosinophilic cationic protein (ECP) levels in tissue homogenates were measured using commercial ELISA kits (Feiya Biological Technology, Jiangsu, China). The protein levels of 34 inflammatory mediators including cytokines, chemokines, and immunoglobulins were quantified with the Bio-Plex suspension chip method (Bio-Rad, Hercules, Calif, United States) (Liao et al., 2018). Concentrations of detected mediators were normalized to total tissue protein levels. More information is provided in this article’s Online Supplement including Supplementary Table SE5.

### Classification of NPs Based on Type 1, 2 and 3 Cytokines

NPs were classified into T1, T2, and T3 endotypes when the protein levels of IFN-γ, IL-5 and IL-17 A were higher than the 95th percentile of the corresponding cytokine levels in control tissues (cut-off value), as previously described (Tomassen et al., 2016; Chen et al., 2021). When a sample showed levels of two or three of these cytokines above the cut-off values, it was considered as a double or triple mixed type. A sample was defined as all negative when the levels of all three cytokines were below the cut-off values.

### Peripheral Blood Neutrophil Isolation and Culture

Neutrophils were purified from peripheral blood of healthy subjects as previously described (Wilkinson et al., 2018; Grunwell et al., 2019). The purity of isolated CD16^+^Siglec-8^-^ neutrophils was greater than 97% as determined by flow cytometry (Supplementary Figure SE1). 1×10^6^ neutrophils were incubated with 100 μg/ml homogenates of control tissues, Neu-high NPs, or Neu-low NPs at 37°C for 8 h. Anti-G-CSF (2 μg/ml, R and D Systems, Minneapolis, MN, United States), anti-IL-6 (2 μg/ml, R and D Systems), or isotype control IgG (2 μg/ml, R and D Systems) (Supplementary Table SE6) was added to certain culture experiments with homogenates of same Neu-high NP samples. Apoptotic, live, and necrotic neutrophils were analyzed by flow cytometry after culture. More information is provided in this article’s Online Supplement.

### Flow Cytometry

The flow cytometric analysis of blood and cultured neutrophils was performed as previously described (Wilkinson et al., 2018). Please refer to the Online Supplement for more information.

### Statistical Analysis

Kolmogorov-Smirnov test or Shapiro-Wilk test was used to test the normality of the data distribution. As all data were non-normally distributed, a Kruskal-Wallis test with the Dunn *post hoc* test was used to adjust for multiple comparisons, and binary comparisons were carried out with a Mann-Whitney *U* 2-tailed. Cell culture data are analyzed by Friedman test or Kruskal-Wallis test, multiple comparisons were carried out using Dunn *post hoc* test. Unless otherwise defined, data are shown as dot plots with horizontal bars representing the medians and error bars showing the interquartile ranges. The correlations were analyzed with a non-parametric Spearman correlation test. For categorical variables, a chi-square test or Fisher’s exact test was used to assess the difference between groups. A logistic regression model was applied to identify risk factors associated with difficult-to-treat disease. SPSS version 22.0 (SPSS Inc., Chicago, IL, United States) software was used to perform the above statistical analyses. In addition, principal component analysis (PCA) and multiple correspondence analysis (MCA) were performed using the R package “devtools” and “MASS,” respectively. Unsupervised hierarchical clustering of variables (Spearman correlation) was performed using R package “pheatmap,” and represented as a dendrogram. A *p* value of less than 0.05 was considered significant.

## Results

### Tissue Neutrophil Distribution in Control Subjects and CRSwNP Patients

**FIGURE 1:**
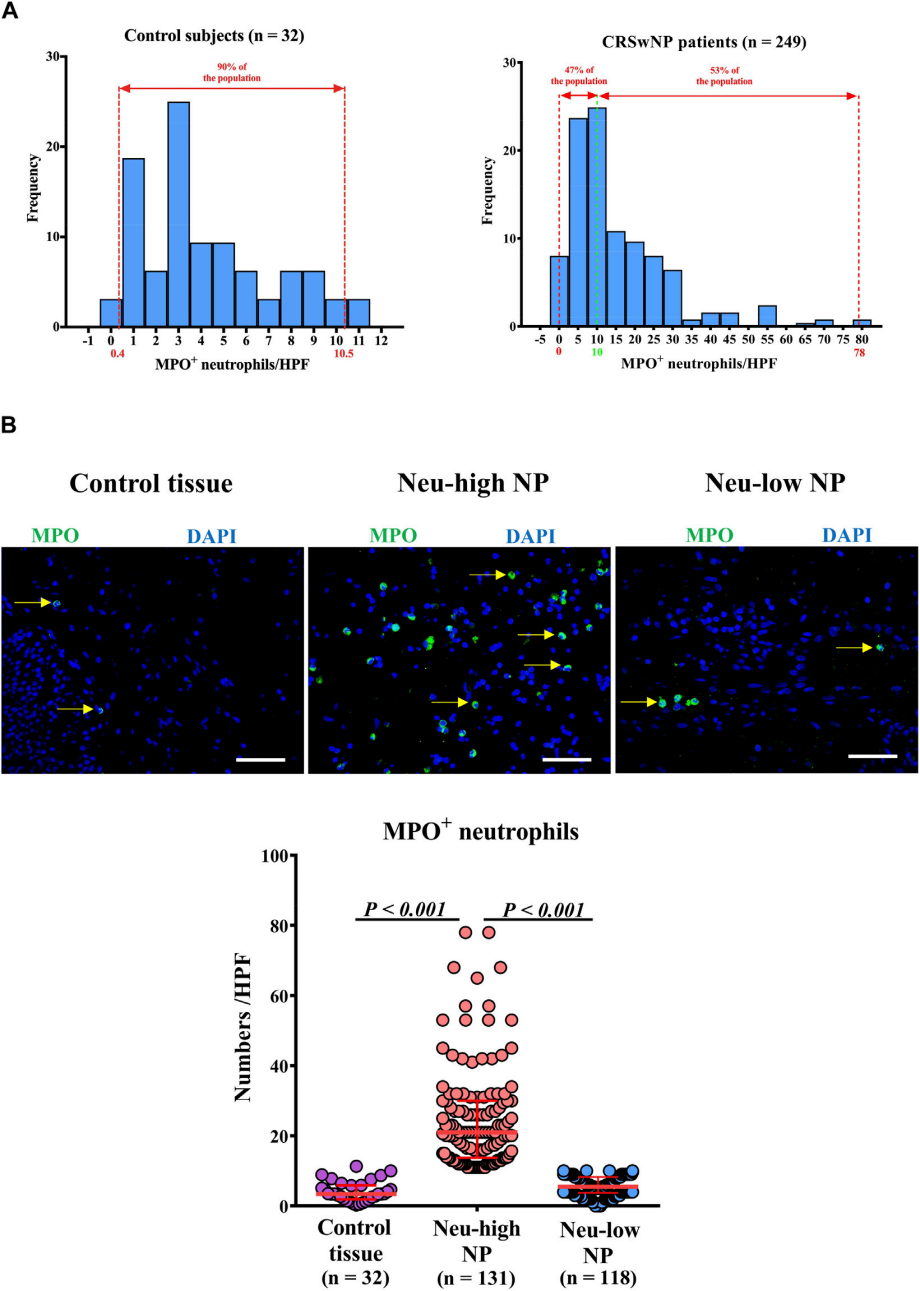
Distribution of tissue neutrophils in patients with CRSwNP. **(A)** Distribution of tissue neutrophils in control subjects **(right panel)** and CRSwNP patients **(left panel)**. The red dotted lines represent 5^th^ percentile (0.4) and 95th percentile (10.5) in the left panel. The red dotted lines represent minimum (0) and maximum (78.0), and the green dotted line represents the cut-off value (10) to define Neu-low and Neu-high NPs in the right panel. **(B)** Representative photomicrographs showing immunofluorescence staining of MPO positive cells and quantification of MPO positive cells. Original magnification ×400. Scale bar, 100 μm. Arrows denote representative positive cells. CRSwNP, chronic rhinosinusitis with nasal polyps; NP, nasal polyp; Neu-high, neutrophil-high; Neu-low, neutrophil-low; MPO, myeloperoxidase; HPF, high-power field.

We first investigated the tissue neutrophil distribution in control subjects and CRSwNP patients. We found that control inferior turbinate and control ethmoid sinus mucosal samples had comparable numbers of MPO^+^ neutrophils and MPO levels (Supplementary Figures SE2A,B). Given the larger amount of samples generally obtained from inferior turbinates, we used inferior turbinate tissues as a control for further study. We found that the numbers of MPO^+^ neutrophils/HPF were not normally distributed in either control or CRSwNP subjects (Figure 1A). The numbers of neutrophils/HPF in control subjects ranged from 0 to 11.3, with a median of 3.4, 5^th^ percentile of 0.4, and 95th percentile of 10.5 (Figure 1A). The numbers of neutrophils/HPF in NPs ranged from 0 to 78.0, with a median of 11.0, 5^th^ percentile of 2.0, and 95th percentile of 45.0 (Figure 1A). We used the 95th percentile of tissue neutrophil numbers in control subjects (>10 MPO^+^ cells/HPF) to define Neu-high NPs. We found that 53% (131/249) of NPs were classified as Neu-high NPs (Figure 1A) and the numbers of MPO^+^ neutrophils were significantly higher in Neu-high NPs than in control tissues and Neu-low NPs (Figure 1B). To further validate this stratification, we analyzed MPO levels in nasal tissues in another set of patients and found that MPO levels were significantly increased in Neu-high NPs in comparison with Neu-low NPs and control tissues (Supplementary Figure SE3A).

### Clinical Characteristics of Patients With Neu-Low NPs and Neu-High NPs

Patients with Neu-high NPs had a lower frequency of asthma comorbidity than patients with Neu-low NPs (Table 1). There was no significant difference regarding age, gender, atopy, allergic rhinitis comorbidity, prior surgery, or disease duration between these two patient groups (Table 1). Compared to patients with Neu-low NPs, patients with Neu-high NPs had milder smell dysfunction, lower posterior ethmoid sinus CT scores, and lower blood eosinophil counts and percentages (Table 1). There was no difference in endoscopic scores, including NP scores, between patients with Neu-low and Neu-high NPs.

**TABLE 1:** Demographic and clinical characteristics of patients with Neu-high and Neu-low NPs.

	Patients with Neu-high NPs (*n* = 131)	Patients with Neu-low NPs (*n* = 118)	*p* Value
Gender, male, N (%)	90 (69%)	76 (64%)	0.473
Age (years)	40.0 (25.0–51.0)	44.0 (34.5–51.0)	0.133
Patients with atopy	22 (17%)	27 (23%)	0.228
Patients with AR	16 (12%)	15 (13%)	0.905
Patients with asthma	9 (7%)	18 (15%)	**0.034**
Disease duration (y)	4.00 (1.00–10.00)	5.00 (2.00–10.00)	0.401
Patients with prior surgery	44 (34%)	45 (38%)	0.455
Symptom VAS score			
Nasal obstruction	7.00 (5.00–8.00)	7.00 (5.00–9.00)	0.471
Rhinorrhea	5.00 (3.00–8.00)	5.00 (2.00–7.00)	0.128
Headache	2.00 (0.00–5.00)	2.50 (0.00–5.00)	0.442
Facial pain	0.00 (0.00–2.50)	0.00 (0.00–2.00)	0.863
Loss of smell	5.00 (1.00–8.00)	8.00 (3.25–10.00)	**0.015**
Total score	22.00 (15.00–29.00)	19.50 (14.00–26.00)	0.068
Overall burden	7.00 (5.00–8.00)	6.00 (5.00–8.00)	0.880
Endoscopic score			
Polyp	4.00 (2.00–5.00)	3.00 (2.00–4.00)	0.235
Edema	2.00 (1.00–2.00)	2.00 (1.50–2.00)	0.863
Discharge	2.00 (1.00–2.00)	2.00 (0.75–2.00)	0.435
Scarring	0.00 (0.00–0.00)	0.00 (0.00–0.00)	0.758
Crusting	0.00 (0.00–0.00)	0.00 (0.00–1.25)	0.634
Total score	8.00 (6.00–11.00)	9.00 (8.00–10.00)	0.254
Bilateral CT score			
Frontal sinus	1.00 (0.00–3.50)	2.00 (0.00–4.00)	0.734
Anterior ethmoid sinus	3.00 (2.00–4.00)	4.00 (2.25–4.00)	0.231
Posterior ethmoid sinus	2.00 (1.00–4.00)	4.00 (2.00–4.00)	**0.048**
Maxillary sinus	2.00 (1.50–4.00)	2.50 (2.00–4.00)	0.384
Sphenoid sinus	1.00 (0.00–3.00)	2.00 (0.00–3.50)	0.257
OMC	3.00 (2.00–4.00)	4.00 (3.00–4.00)	0.152
Total score	15.00 (9.00–22.00)	17.50 (12.00–21.00)	0.384
Blood routine			
Blood leukocyte count (×10^9^)	5.68 (4.98–6.80)	6.19 (5.65–7.37)	0.058
Blood neutrophil count (×10^9^)	3.36 (2.65–3.86)	3.19 (2.65–4.21)	0.287
Blood neutrophil percent (%)	51.80 (47.70–57.60)	55.15 (47.70–60.70)	0.286
Blood lymphocyte count (×10^9^)	2.02 (1.64–2.45)	2.10 (1.78–2.46)	0.195
Blood lymphocyte percent (%)	34.10 (29.80–41.45)	32.00 (26.93–38.18)	0.072
Blood eosinophil count (×10^9^)	0.19 (0.10–0.28)	0.29 (0.13–0.56)	**< 0.001**
Blood eosinophil percent (%)	3.10 (1.60–4.60)	4.70 (1.93–7.90)	**0.002**
Blood monocyte count (×10^9^)	0.46 (0.36–0.55)	0.49 (0.40–0.63)	0.394
Blood monocyte percent (%)	7.50 (6.40–9.20)	7.60 (6.20–8.90)	0.734

NPs, nasal polyps; Neu-high, neutrophil-high; Neu-low, neutrophil-low; AR, allergic rhinitis; VAS, visual analogue scale; CT, computed tomography; OMC, ostiomeatal complex. For continuous variables, results are expressed as medians and interquartile ranges. Categorical variables are summarized using frequency and percentage. The *p* values in bold indicate the values less than 0.05.

### Immunopathological Features of Neu-High and Neu-Low NPs

By evaluating HE-stained tissue sections, we found that Neu-low NPs had higher numbers of eosinophils than Neu-high NPs and control tissues, and there was no difference between Neu-high NPs and control tissues (Supplementary Table SE7). Although both Neu-low and Neu-high NPs had higher numbers of plasma cells and mononuclear cells than in control tissues, there was no difference between Neu-low and Neu-high NPs (Supplementary Table SE7). To confirm the results of eosinophil infiltration, we analyzed the ECP levels in tissues in another set of patients and found that Neu-low NPs had increased ECP levels compared to Neu-high NPs and control tissues (Supplementary Figure SE3B).

**FIGURE 2:**
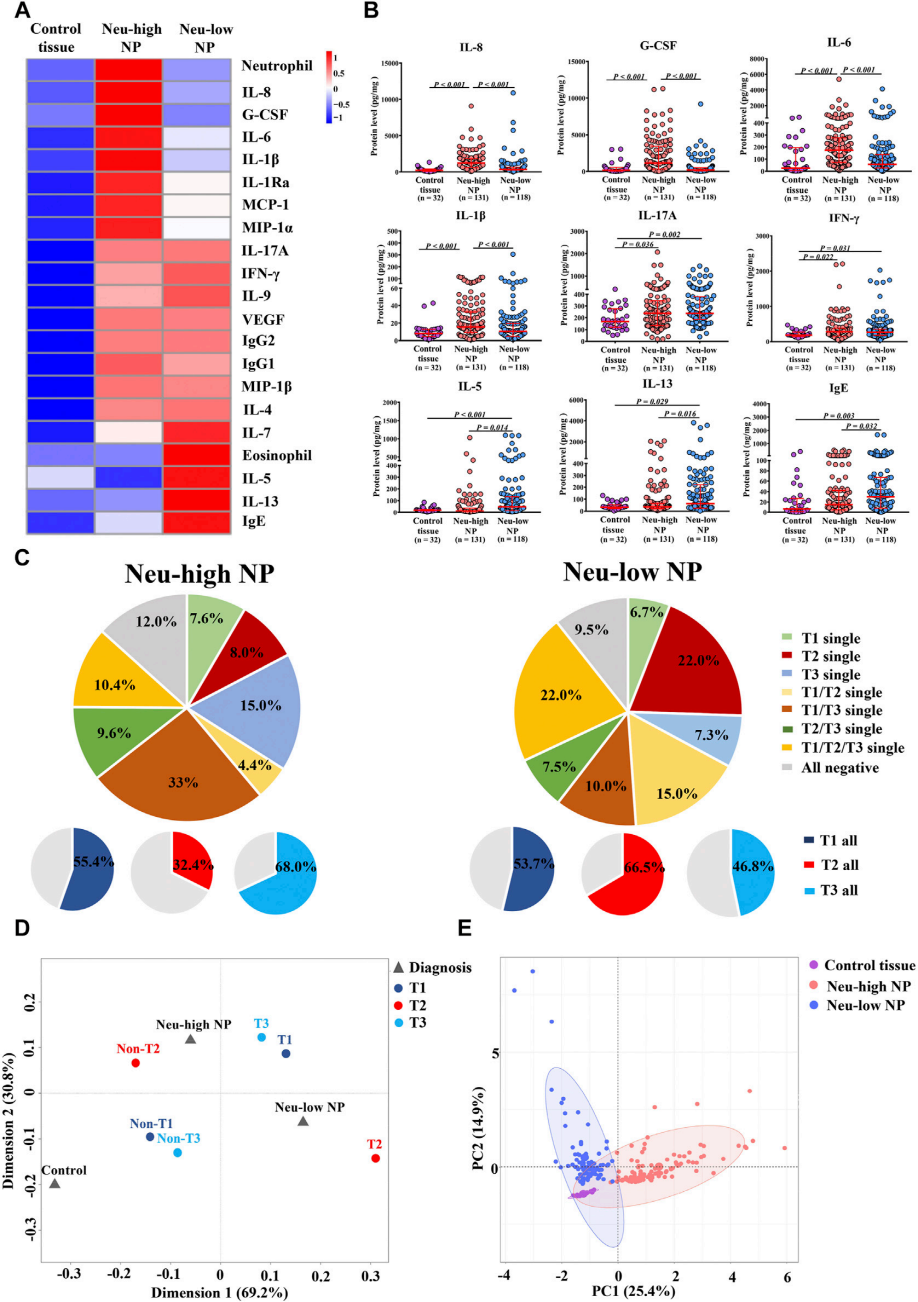
Inflammatory features of Neu-high and Neu-low NPs. **(A)** Heatmap showing inflammatory mediators with different expression in at least one of the three groups as compared to other groups along with neutrophil and eosinophil numbers. **(B)** The levels of selected inflammatory mediators. **(C)** Patterns of T1, T2, and T3 endotype in Neu-high NPs **(left panel)** and Neu-low NPs **(right panel)**. **(D)** Multiple correspondence analysis plot for the interrelationships between Neu-high NP, Neu-low NP, control tissue, and T1, T2 and T3 endotype. **(E)** Principal component analysis based on inflammatory mediators and cells indicated in heatmap. NP, nasal polyp; Neu-high, neutrophil-high; Neu-low, neutrophil-low; G-CSF, granulocyte colony-stimulating factor; IFN-γ, interferon γ; Ig, immunoglobulin; IL, interleukin; IL-1Ra, IL-1 receptor antagonist; MCP-1, monocyte chemoattractant protein-1; MIP, macrophage inflammatory protein; VEGF, vascular endothelial growth factor.

Using the Bio-Plex suspension chip method, we measured the protein levels of 34 inflammatory mediators in sinonasal tissues (Supplementary Table SE8). Those having different expression in at least 1 of the 3 subject groups as compared to other groups are shown in the heatmap in Figure 2A, along with tissue neutrophil and eosinophil numbers. The expression levels of selected mediators are shown in Figure 2B. Neu-high NPs displayed increased levels of neutrophil-relevant cytokines and chemokines, including G-CSF, IL-8, IL-6, MCP-1, MIP-1α, IL-1β, and IL-1Ra compared with Neu-low NPs and control tissues (Figures 2A,B). In contrast, the levels of eosinophilic inflammation associated markers including IL-5, IL-13, and IgE were higher in Neu-low NPs than in Neu-high NPs and control tissues (Figures 2A,B). The levels of IFN-γ and IL-17A were increased in both Neu-low and Neu-high NPs compared with control tissues and no significant difference between Neu-high and Neu-low NPs was found (Figures 2A,B).

We next classified Neu-high and Neu-low NPs into T1, T2, and T3 endotypes based on tissue levels of IFN-γ, IL-5, and IL-17A, respectively. We found that the T1, T2, and T3 endotypes accounted for 55.4, 32.4, and 68.0% of Neu-high NPs, respectively, which was distinct from Neu-low NPs (53.7, 66.5, and 46.8% for the T1, T2, and T3 endotypes, respectively) (Figure 2C). Further MCA demonstrated that Neu-high NPs were located near non-T2 and T3 endotypes, whereas the Neu-low NPs were situated in the middle of T1 and T2 endotypes (Figure 2D). We also performed PCA analysis based on the 21 biomarkers listed in the heatmap, and found that control tissues, Neu-high NPs, and Neu-low NPs were mostly separated from each other (Figure 2E). These comprehensive analyses suggest Neu-high and Neu-low NPs have distinct immunopathological features.

### Molecular Factors Associate With Tissue Neutrophilia in NPs

**FIGURE 3:**
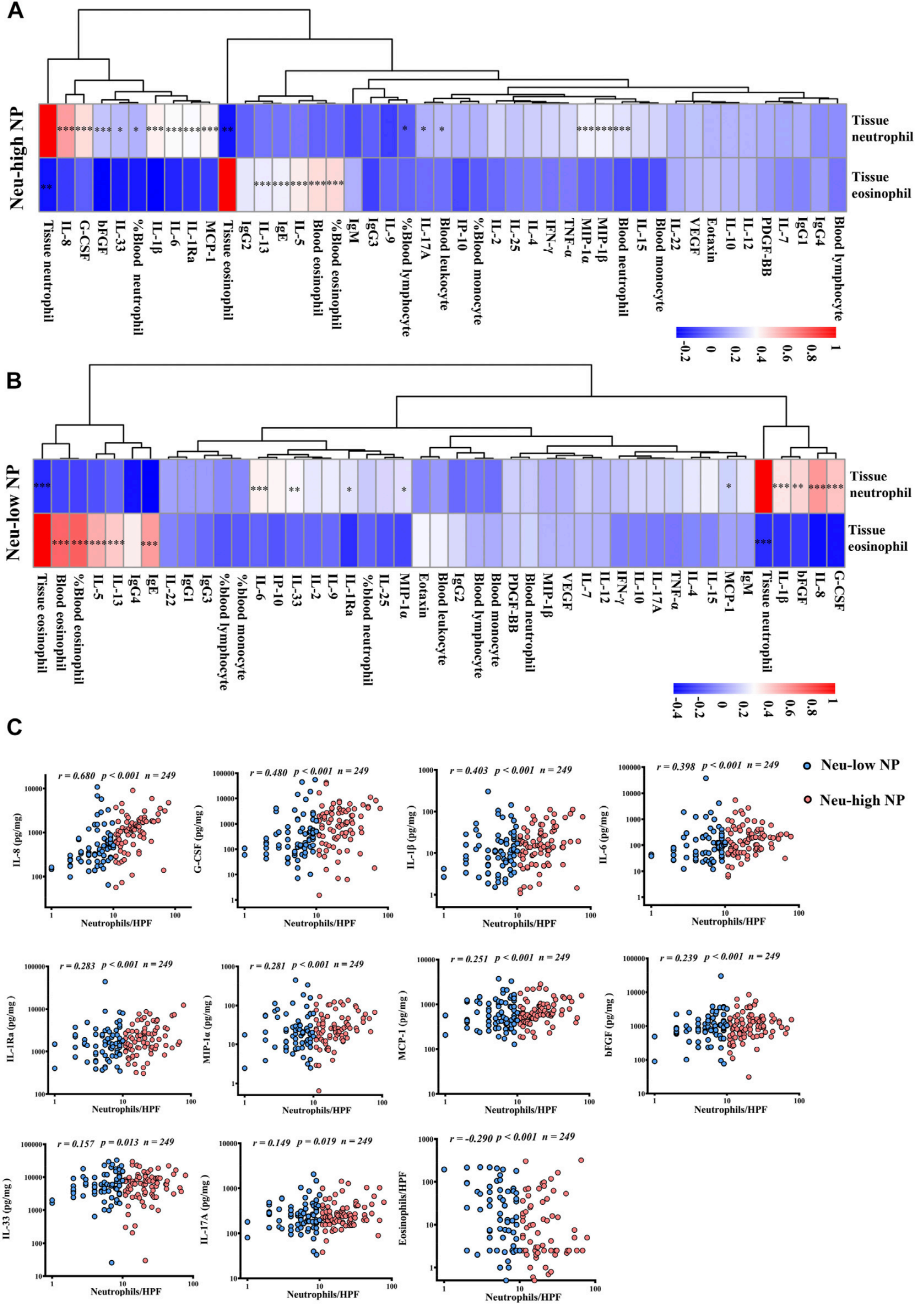
Correlations between tissue neutrophils, inflammatory cells, and mediators in the tissues and peripheral blood of patients with CRSwNP. A and B, Spearman correlation heatmap demonstrates unsupervised hierarchical clustering of the levels of tissue neutrophils and eosinophils, nine blood routine parameters, and 34 tissue inflammatory molecules in Neu-high **(A)** and Neu-low **(B)** NPs. Dendrograms are shown as trees, representing the distance between variables. The correlation matrix shows the positive (red) or negative (blue) correlation of two parameters. Color intensity reflects correlation strength. *p < 0.05, **p < 0.01, and ***p < 0.001. **(C)** Statistically significant correlations of tissue neutrophil numbers with the levels of cells and mediators in tissues of all patients with CRSwNP. NP, nasal polyp; Neu-high, neutrophil-high; Neu-low, neutrophil-low; bFGF, basic fibroblast growth factor; G-CSF, granulocyte colony-stimulating factor; IFN-γ, interferon γ; Ig, immunoglobulin; IL, interleukin; IL-1Ra, IL-1 receptor antagonist; IP-10, interferon-γ-induced protein-10; MCP-1, monocyte chemoattractant protein-1; MIP, macrophage inflammatory protein; PDGF-BB, platelet-derived growth factor-BB; TNF-α, tumor necrosis factor α; VEGF, vascular endothelial growth factor; HPF, high power field.

To explore the factors driving neutrophilia in NPs, we analyzed the correlations of tissue neutrophils and eosinophils with blood leucocytes and tissue inflammatory mediators. By unsupervised hierarchical cluster analysis, we found that IL-8, G-CSF, IL-1β, bFGF, and tissue neutrophil clustered together in both Neu-high NPs and Neu-low NPs, whereas IL-5, IL-13, IgE, IgG4, tissue eosinophil, and blood eosinophil clustered together in Neu-low NPs (Figures 3A,B). We found that the number of tissue neutrophils were positively correlated with the levels of IL-1β, IL-1Ra, IL-6, IL-8, IL-33, bFGF, G-CSF, MCP-1, and MIP-1α in both Neu-high and Neu-low NPs (Figures 3A,B). In contrast, the number of tissue neutrophils was negatively correlated with the number of tissue eosinophils in Neu-high and Neu-low NPs (Figures 3A,B). The blood neutrophil counts, blood total leukocyte counts, blood lymphocyte percentages, blood neutrophil percentages, tissue MIP-1β levels, and tissue IL-17A levels were positively correlated with tissue neutrophil numbers only in Neu-high NPs (Figure 3A). When combining Neu-high and Neu-low NPs together, we found that tissue neutrophil numbers were positively correlated with tissue levels of IL-8, G-CSF, IL-1β, IL-6, IL-1Ra, MIP-1α, MCP-1, bFGF, IL-33, and IL-17 A (Figure 3C); however, the correlations with IL-1Ra, bFGF, MIP-1α, MCP-1, IL-33, and IL-17A were very weak, with *r* values less than 0.3. The tissue neutrophil numbers were weakly and negatively correlated with eosinophil numbers in total NPs (Figure 3C). In addition, tissue levels of IgE, IL-5, and IL-13, and blood eosinophil counts and percentages were positively correlated with tissue eosinophil numbers in both Neu-high and Neu-low NPs (Figures 3A,B).

### Reduced Apoptosis of Neutrophils in Neu-High NPs

**FIGURE 4:**
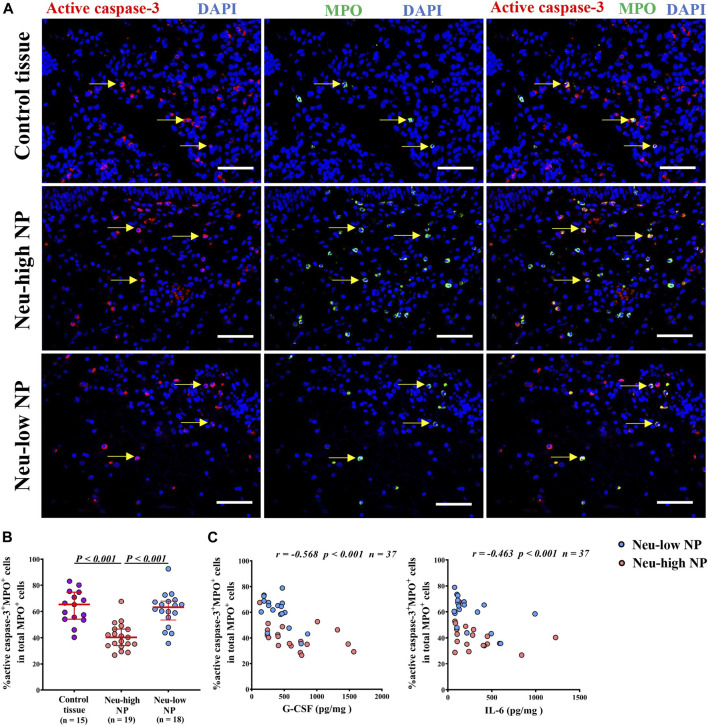
Reduced apoptosis of neutrophils in Neu-high NPs. **(A)** Representative immunofluorescence staining photomicrographs showing active caspase-3^+^MPO^+^ apoptotic neutrophils. Original magnification ×400. Arrows denote representative double positive cells. Scale bar, 100 μm. **(B)** The frequencies of active caspase-3^+^MPO^+^cells in total number of MPO^+^ cells. **(C)** The correlations between G-CSF **(left panel)** and lL-6 **(right panel)** levels and the percentages of active caspase-3^+^MPO^+^ cells in total NP tissues. NP, nasal polyp; Neu-high, neutrophil-high; Neu-low, neutrophil-low; MPO, myeloperoxidase; G-CSF, granulocyte colony-stimulating factor; IL-6, interleukin-6.

Tissue neutrophilia is mainly determined by the amount of recruitment and survival of neutrophils. Increased expression of neutrophil chemokine IL-8 has also been noted in previous studies. (Van Zele et al., 2006; Wang et al., 2016). In contrast, the apoptotic property of neutrophils in NPs remains poorly understood. The increased expression of survival-promoting factors, such as G-CSF, in Neu-high NPs prompted us to further investigate this finding. We found that the percentage of active caspase-3^+^MPO^+^ apoptotic neutrophils in total neutrophils was significantly lower in Neu-high NPs than in control tissues and Neu-low NPs (Figures 4A,B). The representative photomicrographs showing the isotype control staining are presented in Supplementary Figure SE4. In addition, we found that the percentage of apoptotic neutrophils was negatively correlated with the levels of IL-6 and G-CSF in NPs (Figure 4C). We failed to find any correlation of apoptotic neutrophils with other inflammatory mediators tested in NPs (data not shown).

### Neu-High NP Environment Inhibits Neutrophil Apoptosis

**FIGURE 5:**
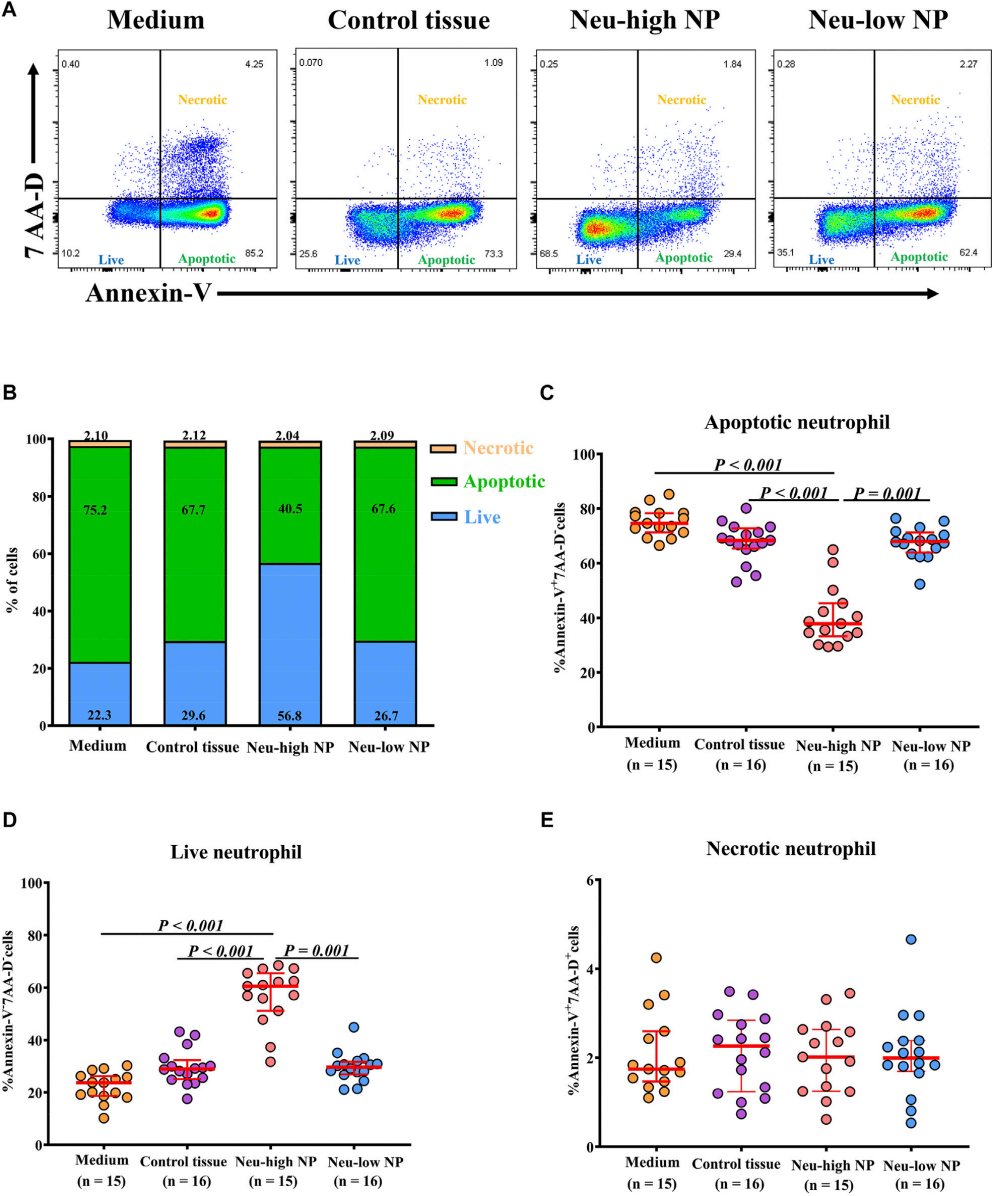
Neu-high NP environment inhibits neutrophil apoptosis. **(A)** Purified blood neutrophils (1 × 10^6^/well) from healthy subjects were cultured in the presence of homogenates (100 μg/ml) of control tissues, Neu-low NPs, and Neu-high NPs for 8 h and then subjected to flow cytometry. Representative flow cytometric analyses of Annexin-V^+^7-AAD^-^ apoptotic, Annexin-V^-^7-AAD^-^ live, and Annexin-V^+^7-AAD^+^ necrotic neutrophils are shown. **(B)** The mean frequencies of apoptotic, live, and necrotic neutrophils in different experimental groups are shown. **(C–E)** The frequencies of Annexin-V^+^7-AAD^-^ apoptotic neutrophils **(C)**, Annexin-V^-^7-AAD^-^ live neutrophils **(D)**, and Annexin-V^+^7-AAD^+^ necrotic neutrophils **(E)** after culture with homogenates of control tissues, Neu-high NPs, and Neu-low NPs. NP, nasal polyp; Neu-high, neutrophil-high; Neu-low, neutrophil-low; AAD, aminoactinomycin D.

Given the observation of reduced neutrophil apoptosis in Neu-high NPs, we hypothesized that the inflammatory milieu in Neu-high NPs could promote the survival of neutrophils. To test this possibility, peripheral blood neutrophils isolated from healthy subjects were cultured with tissue homogenates from the different subject groups. Dexamethasone was used as a positive control. Consistent with a previous study (Cox, 1995), we found that the frequency of Annexin-V^+^7-aminoactinomycin D (AAD)^-^ apoptotic neutrophils was decreased whereas the frequency of Annexin-V^-^7^−^AAD^-^ live neutrophils was increased after dexamethasone treatment when incubated in tissue homogenates of control tissues (Supplementary Figure SE5). Further, we found that the percentage of apoptotic neutrophils was lower whereas the percentage of live neutrophils was higher in neutrophils incubated with tissue homogenates of Neu-high NPs than those incubated with tissue homogenates of control tissues and Neu-low NPs (Figures 5A–E). No difference regarding the frequency of Annexin-V^+^7-AAD^+^ necrotic neutrophils was found among different experimental groups (Figure 5E).

### G-CSF Inhibits Neutrophil Apoptosis in Neu-High NPs

**FIGURE 6:**
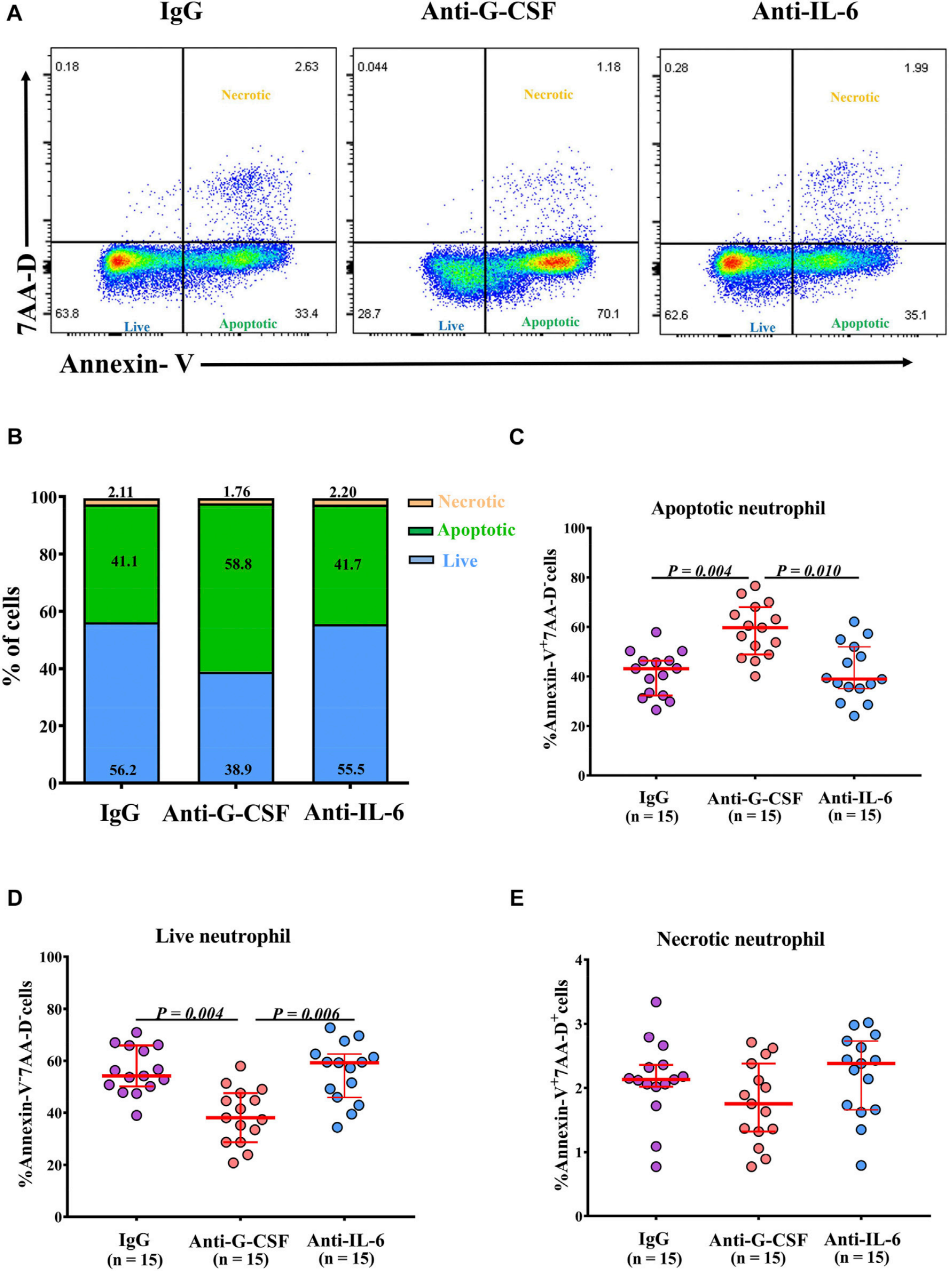
G-CSF inhibits the apoptosis of neutrophils cultured with homogenates of Neu-high NPs. **(A)** Purified blood neutrophils (1 × 10^6^/well) from healthy subjects were cultured in the homogenates of Neu-high NPs in the presence of anti-G-CSF, anti-IL-6, or control IgG. Representative flow cytometric analyses of Annexin-V^+^7-AAD^-^apoptotic, Annexin-V^-^7-AAD^-^ live, and Annexin-V^+^7-AAD^+^ necrotic neutrophils are shown. **(B)** The mean frequencies of apoptotic, live, and necrotic neutrophils in different experimental groups are shown. **(C–E)** The frequencies of Annexin-V^+^7-AAD^-^ apoptotic neutrophils **(C)**, Annexin-V^-^7-AAD^-^ live neutrophils **(D)**, and Annexin-V^+^7-AAD^+^ necrotic neutrophils **(E)** after culture with the homogenates of Neu-high NPs in the presence of anti-G-CSF, anti-IL-6 or control IgG. The data are analyzed in a paired fashion. G-CSF, granulocyte colony-stimulating factor; IL-6, interleukin-6; Ig, immunoglobulin; AAD, aminoactinomycin D; Neu-high, neutrophil-high.

Given the negative correlations between the frequencies of apoptotic neutrophils, G-CSF, and IL-6 levels in NPs, we next explored whether G-CSF or IL-6 was involved in the regulation of neutrophil survival in NPs. We found that the frequency of apoptotic neutrophils was increased, and the frequency of live neutrophils was reduced by anti-G-CSF, but not by anti-IL-6 treatment in neutrophils incubated with tissue homogenates of Neu-high NPs (Figures 6A–E). The frequency of necrotic neutrophils was not affected by either anti-G-CSF or anti-IL-6 treatment (Figure 6E). Next, we detected G-CSF expression in nasal tissues by immunofluorescence staining and found that G-CSF was mainly expressed by epithelial cells. The mean fluorescence intensity of G-CSF staining was significantly higher in Neu-high NPs than that in Neu-low NPs and controls (Supplementary Figure SE6).

### Tissue Neutrophilia Associated With Difficult-To-Treat Disease in Patients With CRSwNP

**FIGURE 7:**
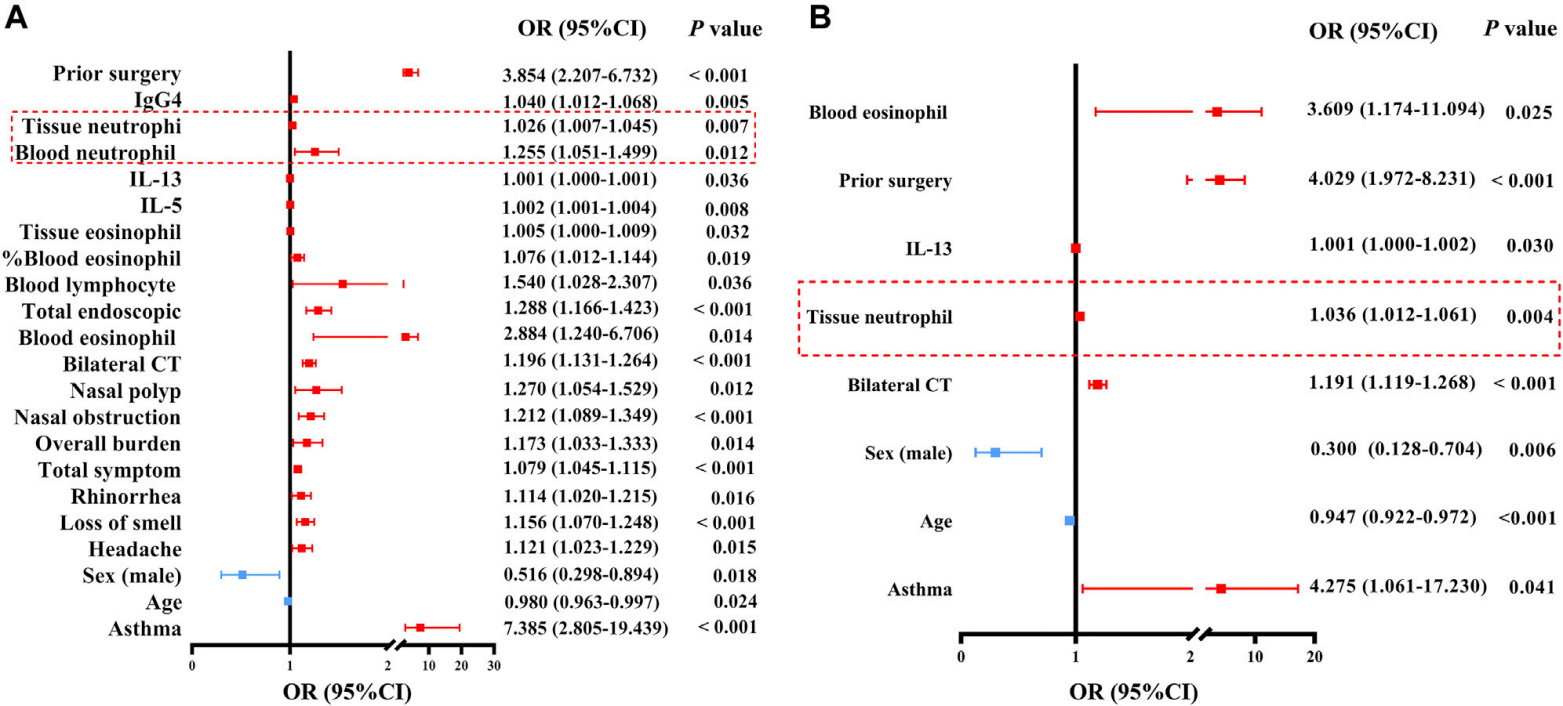
Odds ratios for factors associated with difficult-to-treat CRSwNP. **(A)** Associations between difficult-to-treat disease and clinical features, and cellular and molecular markers, as analyzed via univariate regression analysis in patients with CRSwNP. **(B)** Associations between difficult-to-treat disease and clinical features, and cellular and molecular markers, as analyzed by multivariate regression analysis in patients with CRSwNP. The red frames delineate the neutrophil-associated factors. OR, odds ratio; CI, confidence interval; Ig, immunoglobulin; IL, interleukin; CRSwNP, chronic rhinosinusitis with nasal polyps.

Finally, we performed univariate and multivariate regression analyses to identify clinical, cellular, and molecular factors associated with difficult-to-treat disease in patients with CRSwNP 1 year after surgery (Supplementary Table SE9). Both blood and tissue eosinophil and neutrophil abundance were associated with difficult-to-treat disease using univariate regression analysis (Figure 7A). In addition, the characteristics associated with difficult-to-treat NPs are shown in Figure 7A. When these factors were introduced into a multivariate model, only age, gender, asthma comorbidity, prior surgery, bilateral CT scores, tissue neutrophil counts, blood eosinophil counts, and tissue IL-13 levels were significantly and independently associated with difficult-to-treat disease in patients with CRSwNP (Figure 7B). The risk of difficult-to-treat disease increased by 1.036 times for every one unit increase of tissue neutrophil count, and increased by 3.609 times for every 1×10^9^ increase of blood eosinophil counts.

## Discussion

Although the pathogenic role of neutrophils in CRSwNP has been recognized in patients of both Asian and Western countries, the regulation of tissue neutrophilia in CRSwNP remains poorly studied. In this study, we studied the neutrophil distribution in NPs and control tissues and proposed a cutoff value to define neutrophilic inflammation in NPs by rigorous statistical analysis. We thoroughly compared the difference regarding inflammation patterns, clinical characteristics and treatment outcomes between neutrophil-high NPs and neutrophil-low NPs. We provided evidence of reduced apoptosis of neutrophils in NPs with neutrophilic inflammation for the first time and highlighted the important role of G-CSF in promoting neutrophil survival and tissue neutrophilia in NPs. We also emphasized the negative influence of tissue neutrophilia on treatment outcome in patients with CRSwNP.

To define tissue neutrophilia, we first investigated the tissue neutrophil distribution in control subjects and CRSwNP patients. We found that there was a considerable variation of neutrophil distribution among the CRSwNP patients. Based on the neutrophil infiltration condition in control nasal tissues, we stratified NPs into Neu-low and Neu-high type. This statistical method is a standard method to define cut-off value for pathological condition based on the normal controls (Roccella et al., 1987; Harrington et al., 2013; Thompson et al., 2016). We found that the Neu-high type constituted about half of total NPs. Neutrophilic inflammation had no significant effect on polyp size. However, we found that patients with Neu-low NPs presented a higher frequency of asthma comorbidity, worse smell function, higher CT scores for posterior ethmoid sinus, and higher blood eosinophil counts and percentages. These characteristics have previously been linked to eosinophilic inflammation in NPs (Meng et al., 2016), and such findings suggest that patients with Neu-low NPs could have more prominent eosinophilic inflammation than patients with Neu-high NPs. This observation was confirmed by further comprehensive cellular and molecular analyses. We found that Neu-low NPs were characterized by increased tissue eosinophil numbers and T2 cytokine levels. In addition, 66.5% of Neu-low NPs demonstrated the T2 endotype and Neu-low NPs were located near the T2 on MCA. In contrast, Neu-high NPs were near the non-T2 on MCA and only 32.4% of them had a T2 endotype. Our study indicates a divergence between neutrophilic and eosinophilic inflammation in NPs in Chinese patients. In contrast, Delemarre et al. have recently shown that the number of neutrophils were associated with eosinophil extracellular traps and Chartcot-Leyden crystal levels in NPs in Caucasian patients (Delemarre et al., 2021). Gevaert et al. reported that Charcot-Leyden crystals induced IL-8 production in NP tissues (Gevaert et al., 2020). These studies indicate that eosinophilic inflammation regulates neutrophilic inflammation of NPs in Caucasian patients. The reasons for the differences in eosinophilic and neutrophilic inflammation between Chinese and Caucasian patients with CRSwNP are unclear and require further investigation. In addition, whether Charcot-Leyden crystal formation plays a role in the observed differences between Chinese and Caucasian patients is a topic for future studies.

IL-17 A has been associated with neutrophilic inflammation in the setting of disorders and diseases such as psoriasis (Smolyannikova et al., 2020), asthma (Iwakura et al., 2011), and chronic obstructive pulmonary disease (Roos et al., 2015). However, IL-17A may not have a significant role in regulating neutrophilic inflammation in CRSwNP. Delemarre et al. found that there was no association between IL-17 A levels and the neutrophil infiltration in NPs in Caucasian patients (Delemarre et al., 2021). Saitoh et al. and Makihara et al. found that the numbers of IL-17A positive cells positively correlated with the numbers of eosinophils, but did not correlate with the numbers of neutrophils in Japanese patients with CRSwNP (Makihara et al., 2010; Saitoh et al., 2010). In this study, we found no difference in IL-17A levels between Neu-high and Neu-low NPs, and IL-17A levels had a very weak correlation with tissue neutrophil numbers in NPs. Therefore, these results indicate a lack of potential in using anti-IL-17A biologics to treat neutrophilic inflammation in patients with CRSwNP. Extending previous findings (Reichel et al., 2009; Biondo et al., 2014; Coffelt et al., 2016; de Oliveira et al., 2016; Jorgensen et al., 2016), we found that tissue neutrophils correlated with several neutrophil related chemokines, including IL-1β, IL-8, MCP-1, and MIP-1α. Among them, IL-8 demonstrated the strongest correlation with tissue neutrophils, suggesting a predominant role of IL-8 in recruiting neutrophils to NPs.

In addition to neutrophil chemokines, we found an upregulation of G-CSF and IL-6 in Neu-high NPs compared to Neu-low NPs. Moreover, G-CSF and IL-6 levels positively correlated with tissue neutrophil numbers in NPs. G-CSF and IL-6 have previously been implicated in promoting the survival of neutrophils (Biffl et al., 1995; Droemann et al., 2006; Jun et al., 2011; Dienz et al., 2012). The excessive infiltration of neutrophils in NPs may be due to the anti-apoptotic properties of G-CSF or IL-6. To test this possibility, we investigated the apoptotic properties of neutrophils in NPs. Our study is the first to provide several lines of evidence for a G-CSF role in regulating tissue neutrophilia in NPs: 1) The percentage of apoptotic neutrophils was significantly reduced in Neu-high NPs compared with Neu-low NPs and control tissues; 2) The inflammatory milieu in Neu-high NPs, but not that in Neu-low NPs, inhibited the apoptosis of neutrophils; 3) Blocking G-CSF, but not IL-6, abolished the anti-apoptotic effect of homogenates of Neu-high NPs. Delemarre et al. reported that there was no change in apoptosis of isolated blood neutrophils when cultured with NP tissue homogenates compared to those incubated with control tissue homogenates (Delemarre et al., 2021). However, Delemarre et al. did not take the heterogeneity of neutrophilic inflammation in NPs into consideration, and they also did not detect the apoptotic property of neutrophils in NPs. Our study clearly demonstrates that G-CSF was only elevated in NPs with high neutrophilic inflammation. G-CSF is a hematopoietic growth factor, expressed by various immune and non-immune cells (Roberts, 2005). By binding to CSF3R, a specific G-CSF receptor, G-CSF can prolong the survival of neutrophils and their precursors (Roberts, 2005; Panopoulos and Watowich, 2008). Although G-CSF has typically been linked with the generation of neutrophils in bone marrows, limited studies have explored its role in local inflammatory diseases. To the best of our knowledge, our study is the first to report that G-CSF may promote neutrophil survival, thus contributing to tissue neutrophilia in NPs. In our study, although IL-6 levels negatively correlated with apoptotic neutrophils in NPs, we failed to find a direct anti-apoptotic activity of IL-6 *in vitro*. This suggests that IL-6 may be an indirect factor regulating neutrophil apoptosis. Airway epithelium can be activated by classical IL-6 receptor signaling or IL-6 *trans*-signaling pathways (Jevnikar et al., 2019). We found that nasal epithelial cells were an important source of G-CSF in Neu-high NPs, and IL-6 levels were positively correlated with G-CSF levels in NPs (Supplementary Figure SE7). This suggests that IL-6 may promote G-CSF production in nasal epithelial cells in Neu-high NPs. Although IL-8 induces the migration of neutrophils (Coffelt et al., 2016; de Oliveira et al., 2016), whether IL-8 has an effect on neutrophil survival remains unclear. Li et al. have previously shown that IL-8 promoted endothelial cell survival (Li et al., 2003). Given the strong correlation between IL-8 and neutrophils in NPs, it would be interesting to study whether IL-8 has a role on neutrophil survival in the future.

Previous studies found an association between neutrophilic inflammation and poor treatment outcome in Asian patients with CRSwNP (Liao et al., 2018). To further explore the impact of neutrophilic inflammation on treatment outcome with the consideration of a variety of potential risk factors, we performed univariate and multivariate regression analyses of a number of demographic, clinical, cellular, and molecular factors. We found that tissue neutrophil counts and blood eosinophil counts were independent risk factors for difficult-to-treat disease in patients with CRSwNP 1 year after surgery. Nevertheless, tissue eosinophil counts were not identified as independent risk factors by multivariate regression analysis. Recently, Succar et al. found that tissue neutrophil counts, but not eosinophil counts, were associated with baseline SNOT-22 scores in American patients with CRSwNP (Succar et al., 2020). These findings support a pathogenic role for neutrophils in CRSwNP.

One potential limitation of our study is the use of inferior turbinate as a control, since NPs originate from sinus mucosa. However, we found comparable neutrophil infiltration and MPO levels in the ethmoid mucosa and inferior turbinate tissues in a small sample size study. Inferior turbinate tissues have been widely used as a control for NPs in published studies (Tomassen et al., 2016; Liu et al., 2020; Delemarre et al., 2021). More importantly, in this study, we discovered clear differences between Neu-low and Neu-high NPs. Another limitation is that although we found the levels of IL-1β, IL-6, IL-8 and G-CSF positively correlated with tissue neutrophil numbers in NPs, the *r* values of correlations were not high. It suggests that neutrophilic inflammation is unlikely explained completely by one mechanism in NPs, the complex and heterogeneous disease. In addition, our study demonstrated the variance of tissue neutrophil distribution in NPs and proposed a cut-off value to define Neu-high NPs based on the histological study, which may be helpful to identify the neutrophilic inflammation in clinics. However, we didn’t study the phenotype and function heterogeneity of tissue neutrophils in patients with CRSwNP. Arebro et al. have recently shown the existence of three distinct neutrophil subsets, CD16^dim^CD62L^high^ (immature), CD16^high^CD62L^high^ (mature) and CD16^high^CD62L^dim^ (more activated) subset, in CRSwNP patients, with different activation status (Arebro et al., 2019). Therefore, it would be interesting to study the phenotypic heterogeneity of neutrophils in Neu-high and Neu-low NPs by flow cytometric analysis in future, which may shed light on the pathogenic role of neutrophilic inflammation in CRSwNP.

In summary, our study demonstrates the heterogeneity of neutrophilic inflammation in patients with CRSwNP and comprehensively explored the immunopathological and clinical characteristics associated with neutrophilic inflammation in NPs based on a large cohort of patients with CRSwNP. Furthermore, our study identifies G-CSF from a panel of inflammatory mediators as an important factor in suppressing neutrophil apoptosis and promoting tissue neutrophilia in CRSwNP. In a recent study, a neutralizing anti-G-CSFR antibody has been found to block G-CSF-induced neutrophilia in nonhuman primates (Scalzo-Inguanti et al., 2017). It would be of great value to explore the potential benefit of targeting G-CSF in treating Chinese CRSwNP patients with high neutrophilic inflammation in the future. Given the difference in endotypes of CRSwNP across different geographic area and population with distinct ethnic backgrounds, whether our findings in Chinese patients can be generalized into other populations waits for further investigation.

## Data Availability

The raw data supporting the conclusion of this article will be made available by the authors, without undue reservation.
