# Corrigendum: Characterizing the neutrophilic inflammation in chronic rhinosinusitis with nasal polyps

**DOI:** 10.3389/fcell.2024.1450040

**Published:** 2024-07-18

**Authors:** Jian-Wen Ruan, Jie-Fang Zhao, Xue-Li Li, Bo Liao, Li Pan, Ke-Zhang Zhu, Qi-Miao Feng, Jin-Xin Liu, Zi-E. Yu, Jia Song, Hai Wang, Zheng Liu

**Affiliations:** Department of Otolaryngology-Head and Neck Surgery, Tongji Hospital, Tongji Medical College, Huazhong University of Science and Technology, Wuhan, China

**Keywords:** apoptosis, chronic rhinosinusitis with nasal polyps, granulocyte colonystimulating factor, neutrophil, inflammation

In the published article, there was an error in Figure 1B as published. (In the Neu-low NP panel in Figure 1B, an image from Neu-high NP group was mistakenly inserted). The corrected (Figure 1) and its caption appear below.

**FIGURE 1:**
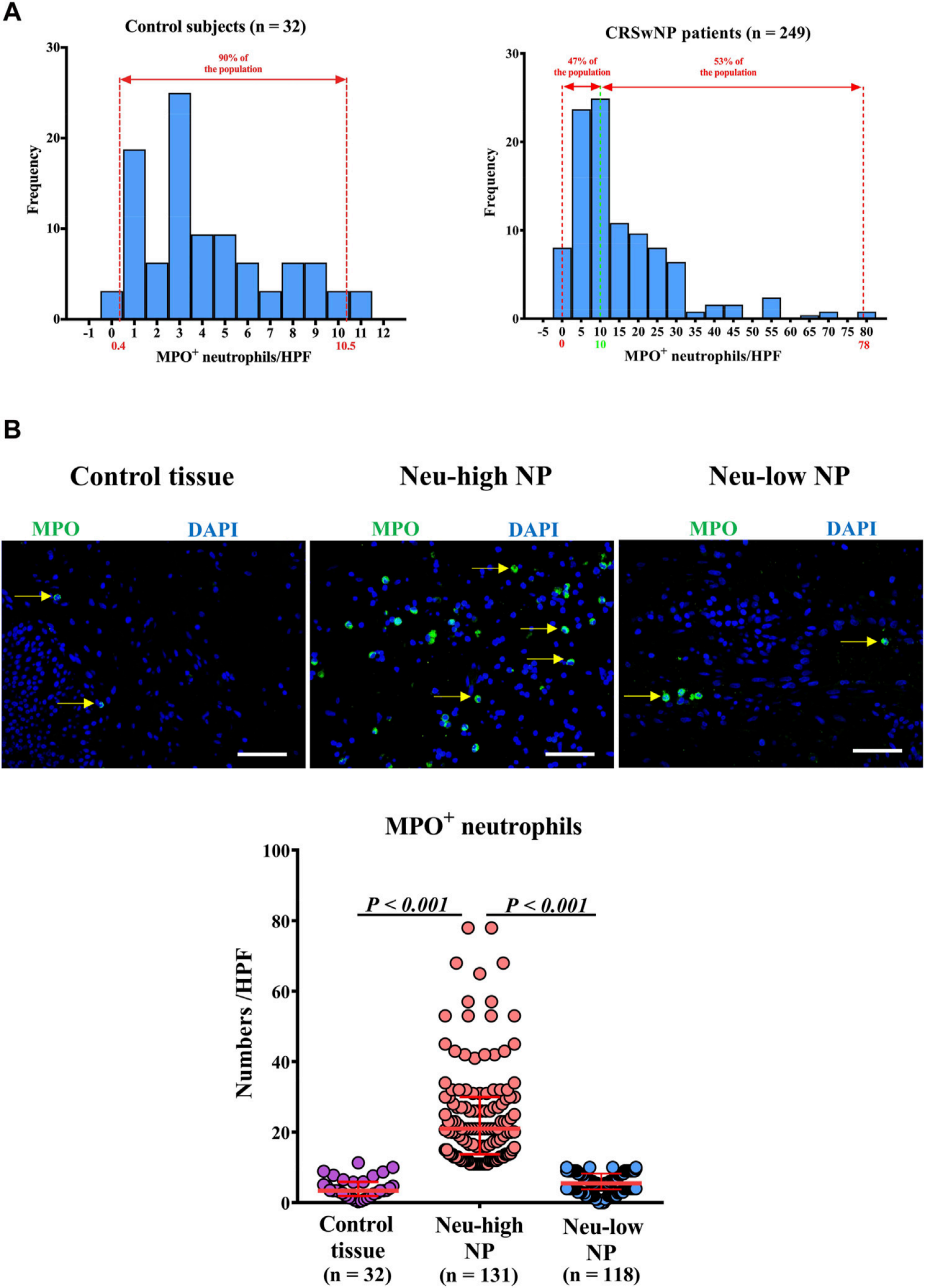
Distribution of tissue neutrophils in patients with CRSwNP. **(A)** Distribution of tissue neutrophils in control subjects (right panel) and CRSwNP patients (left panel). The red dotted lines represent 5th percentile (0.4) and 95th percentile (10.5) in the left panel. The red dotted lines represent minimum (0) and maximum (78.0), and the green dotted line represents the cut-off value (10) to define Neu-low and Neu-high NPs in the right panel. **(B)** Representative photomicrographs showing immunofluorescence staining of MPO positive cells and quantification of MPO positive cells. Original magnification ×400. Scale bar, 100 μm. Arrows denote representative positive cells. CRSwNP, chronic rhinosinusitis with nasal polyps; NP, nasal polyp; Neu-high, neutrophil-high; Neu-low, neutrophil-low; MPO, myeloperoxidase; HPF, high-power field.

The authors apologize for this error and state that this does not change the scientific conclusions of the article in any way. The original article has been updated.

