# Supplementary figures and images for: Characterizing the Neutrophilic Inflammation in Chronic Rhinosinusitis With Nasal Polyps
Source: Front Cell Dev Biol. 2021 Dec 17;9:793073. doi: 10.3389/fcell.2021.793073 (PMC8718617; doi:10.3389/fcell.2021.793073)

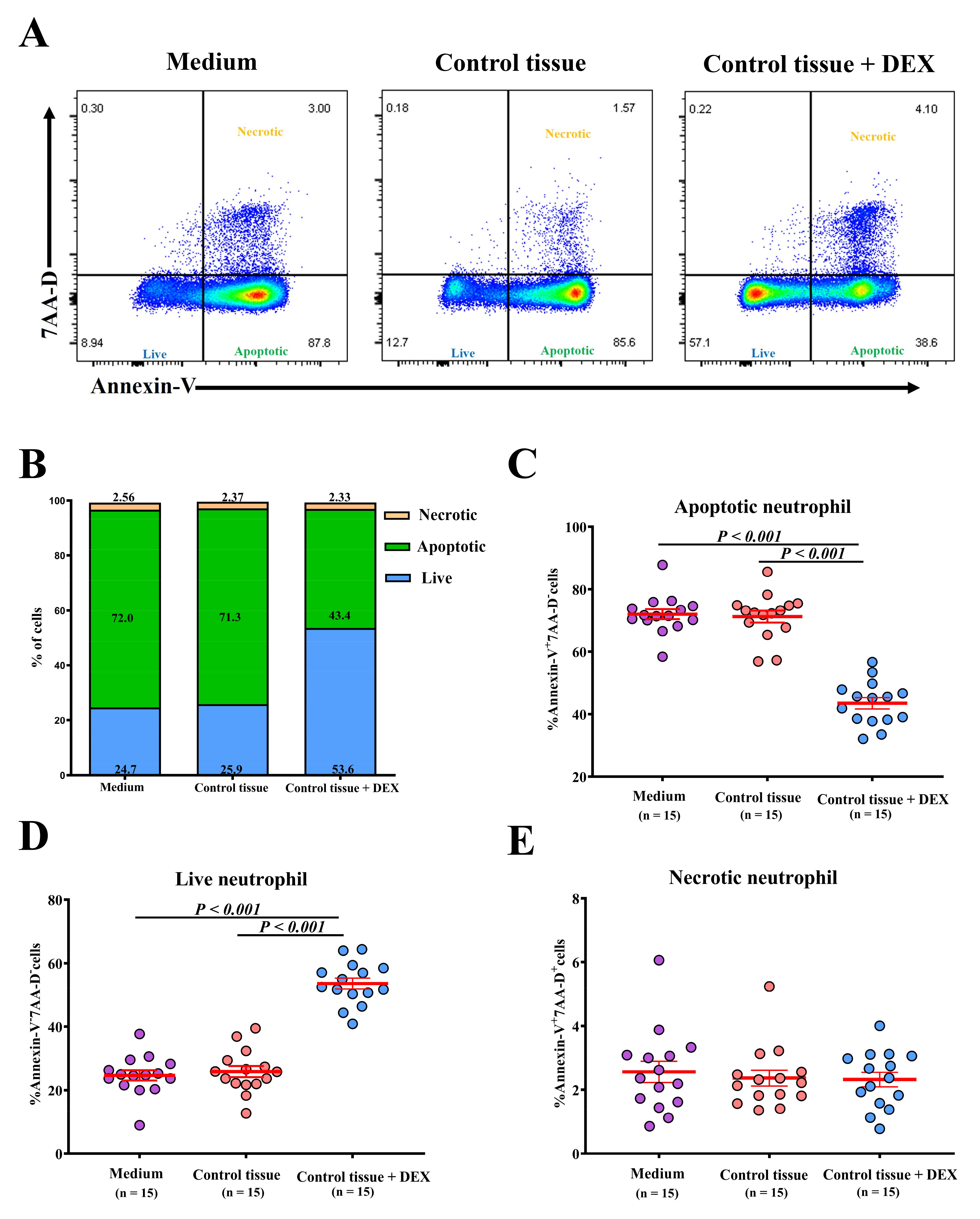

Supplement: Supplementary file 1 [file Image5.jpg]

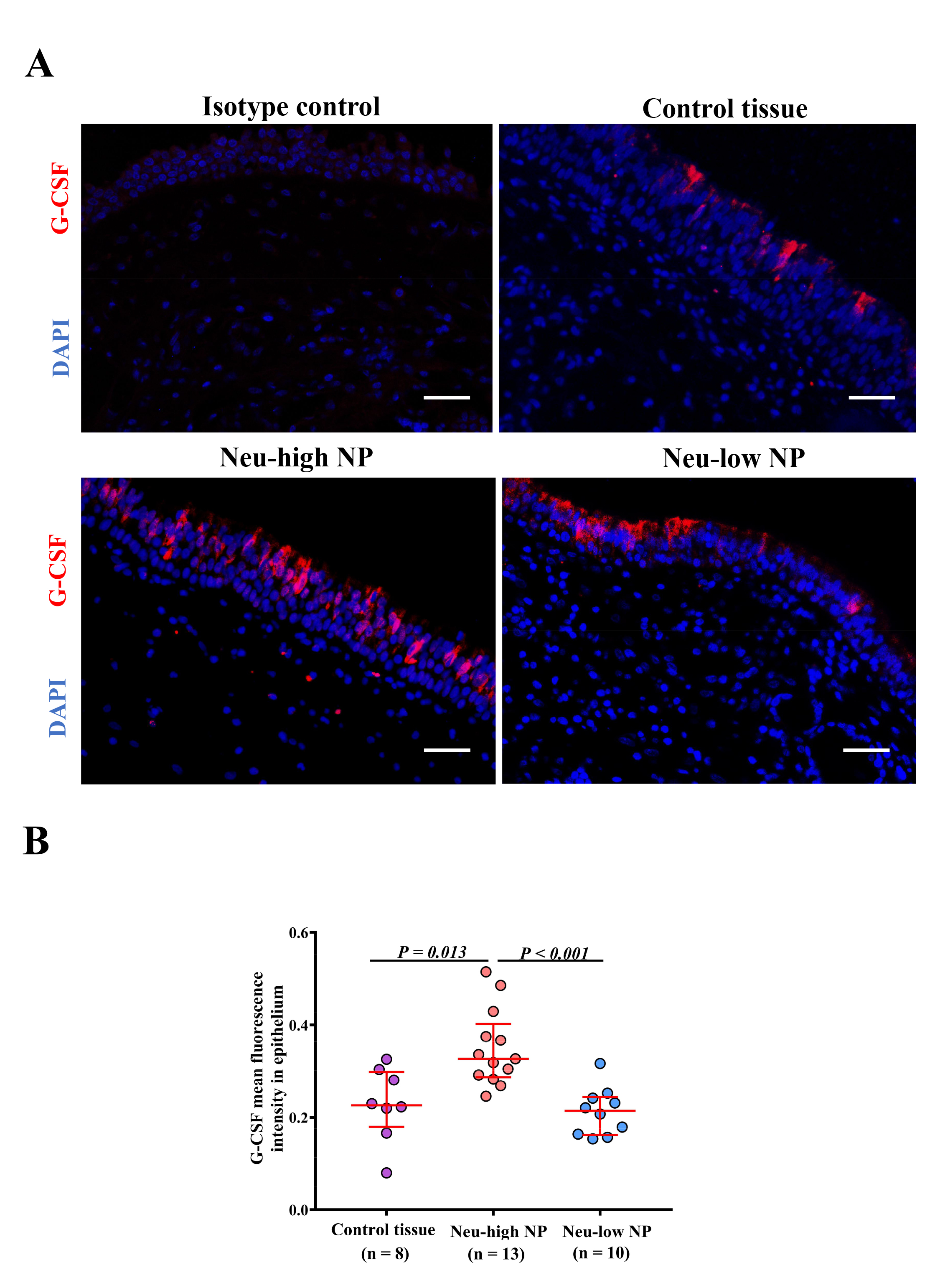

Supplement: Supplementary file 2 [file Image6.jpg]

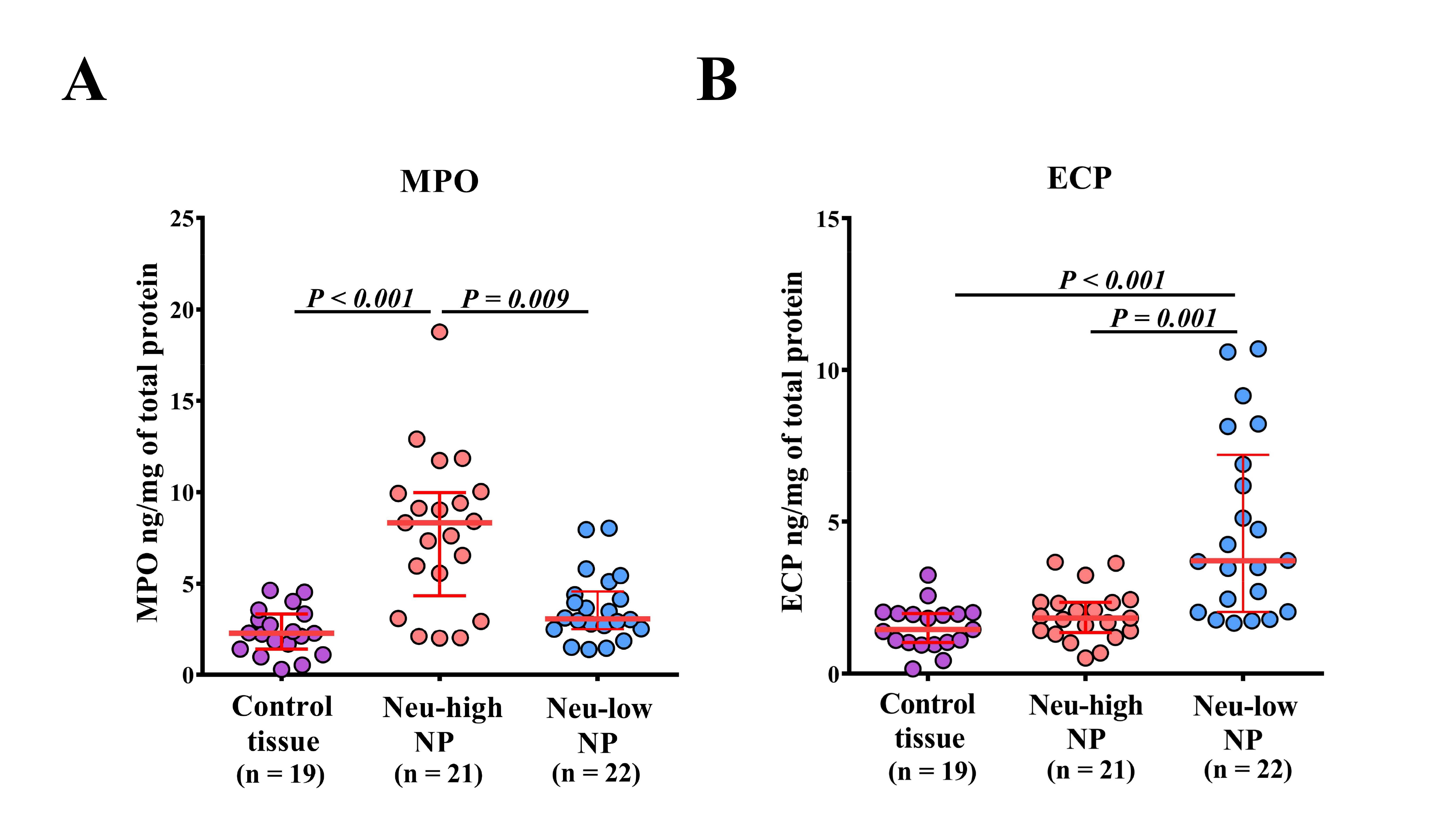

Supplement: Supplementary file 3 [file Image3.jpg]

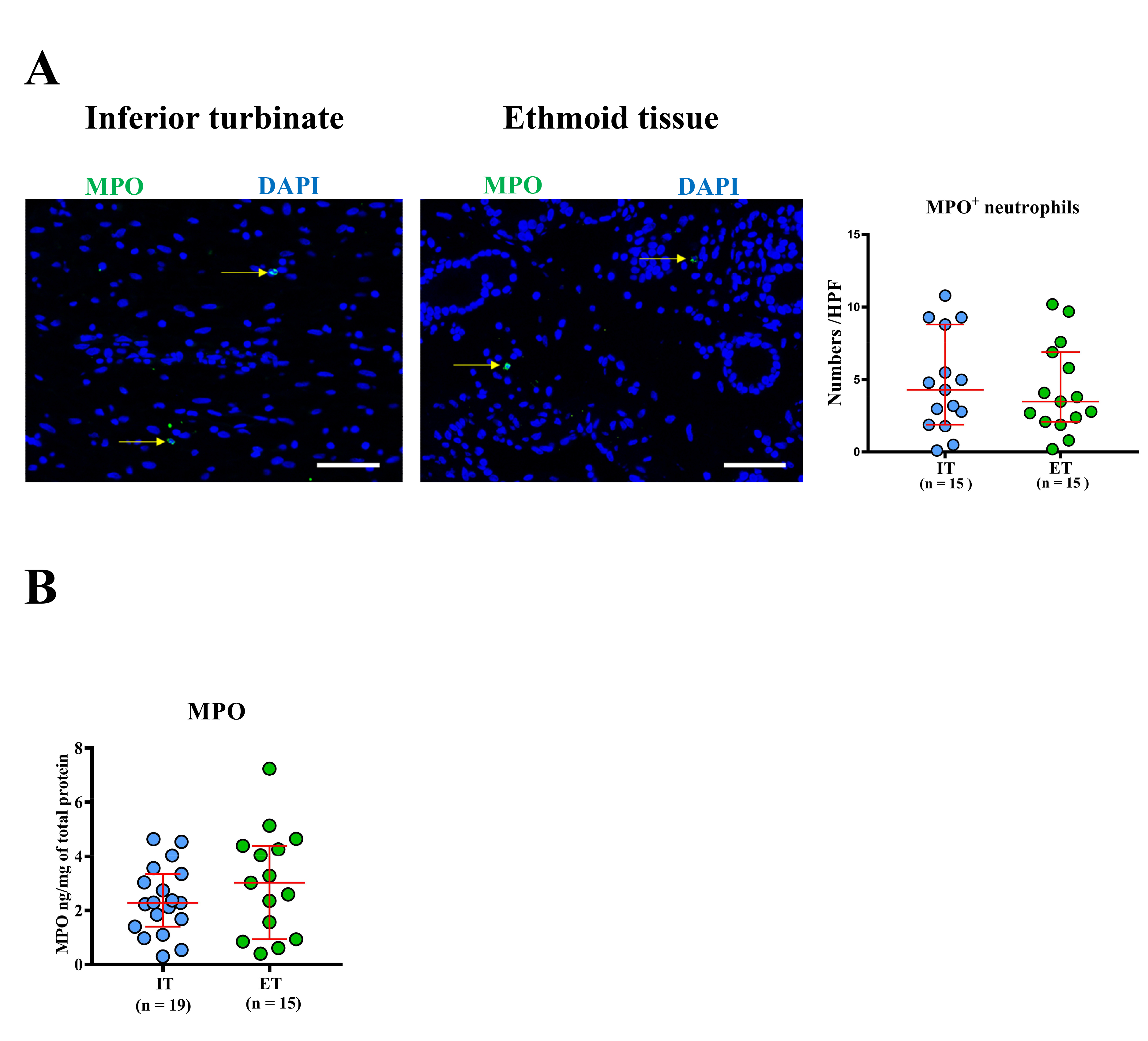

Supplement: Supplementary file 4 [file Image2.jpg]

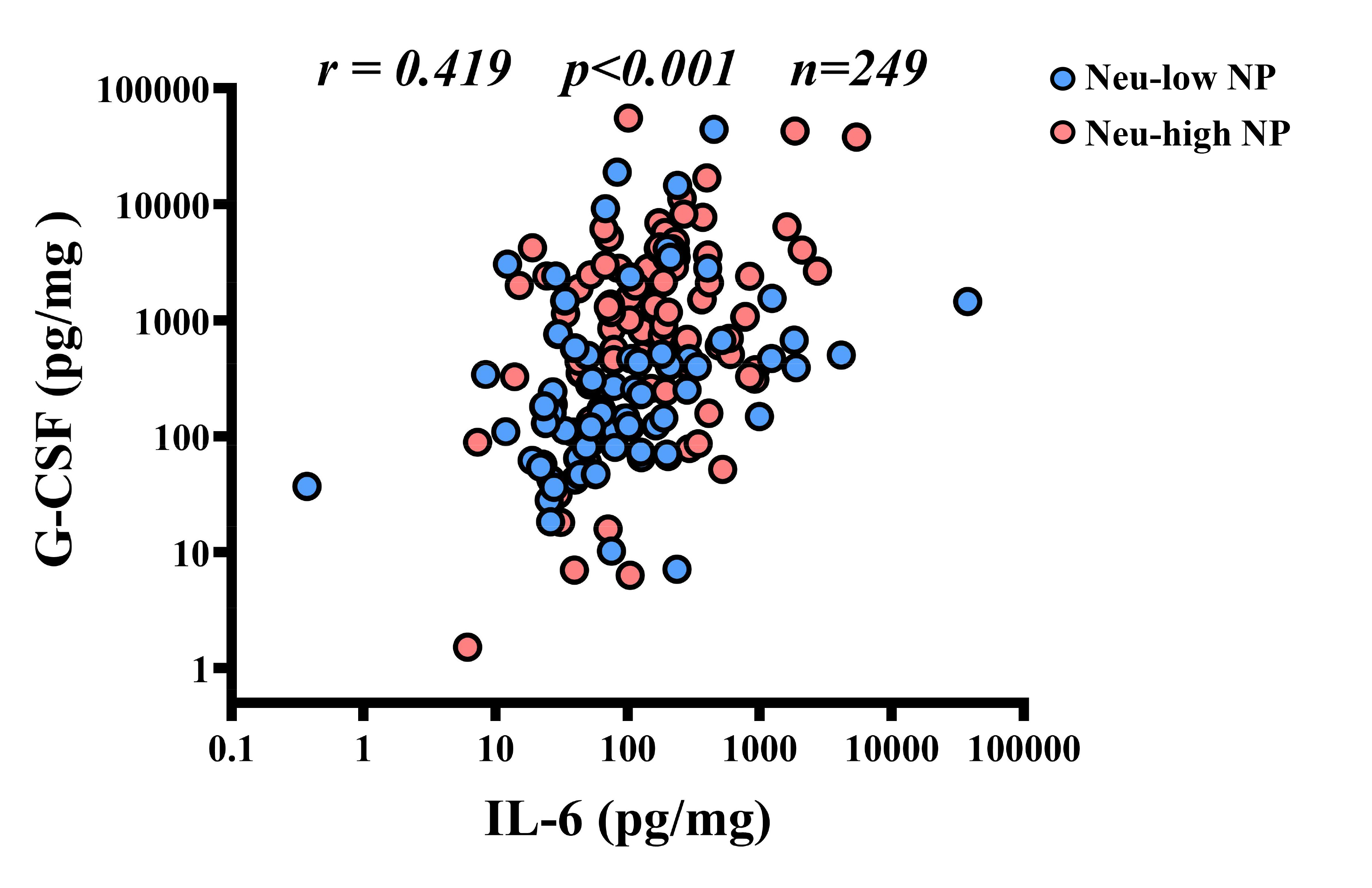

Supplement: Supplementary file 5 [file Image7.jpg]

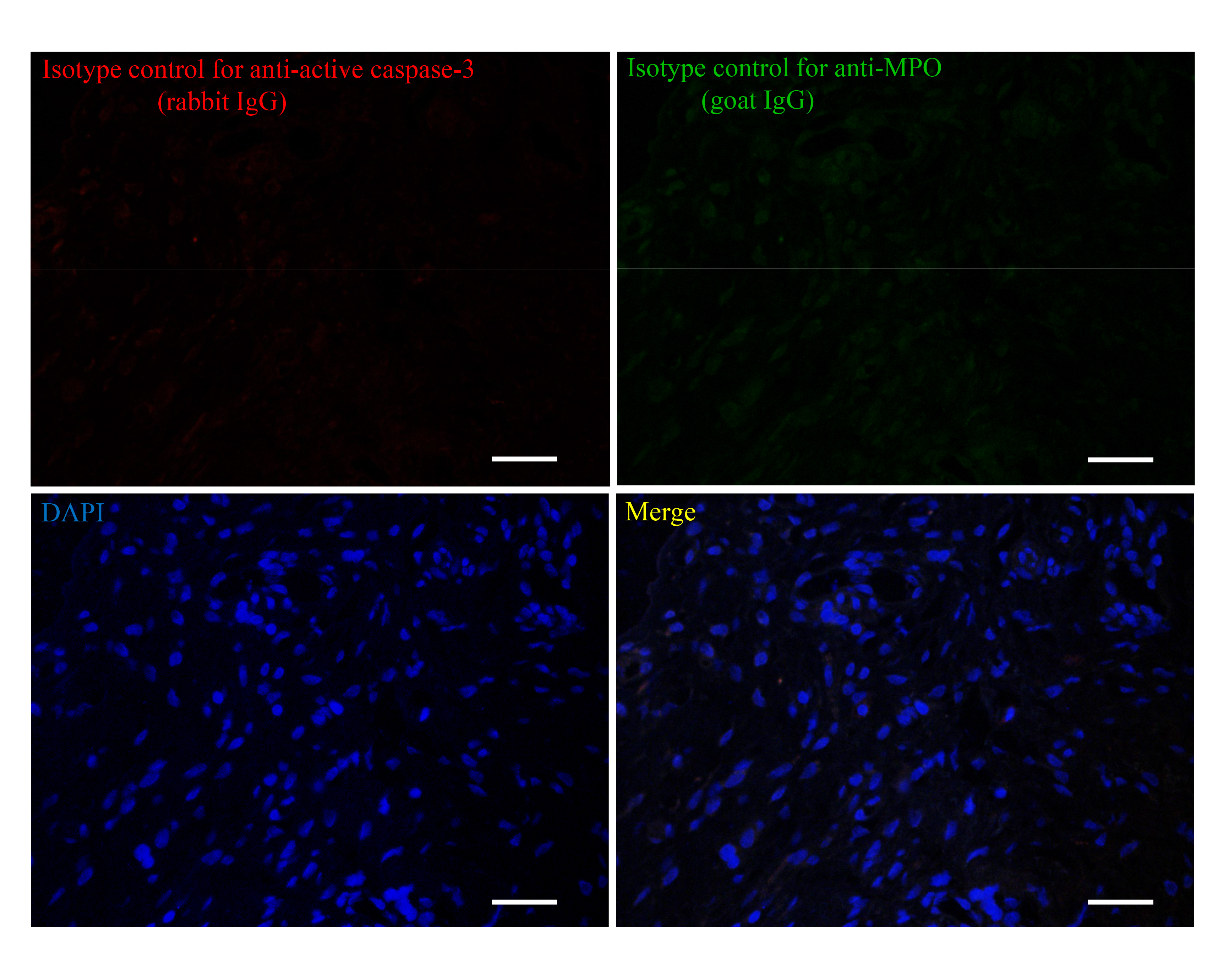

Supplement: Supplementary file 6 [file Image4.jpg]

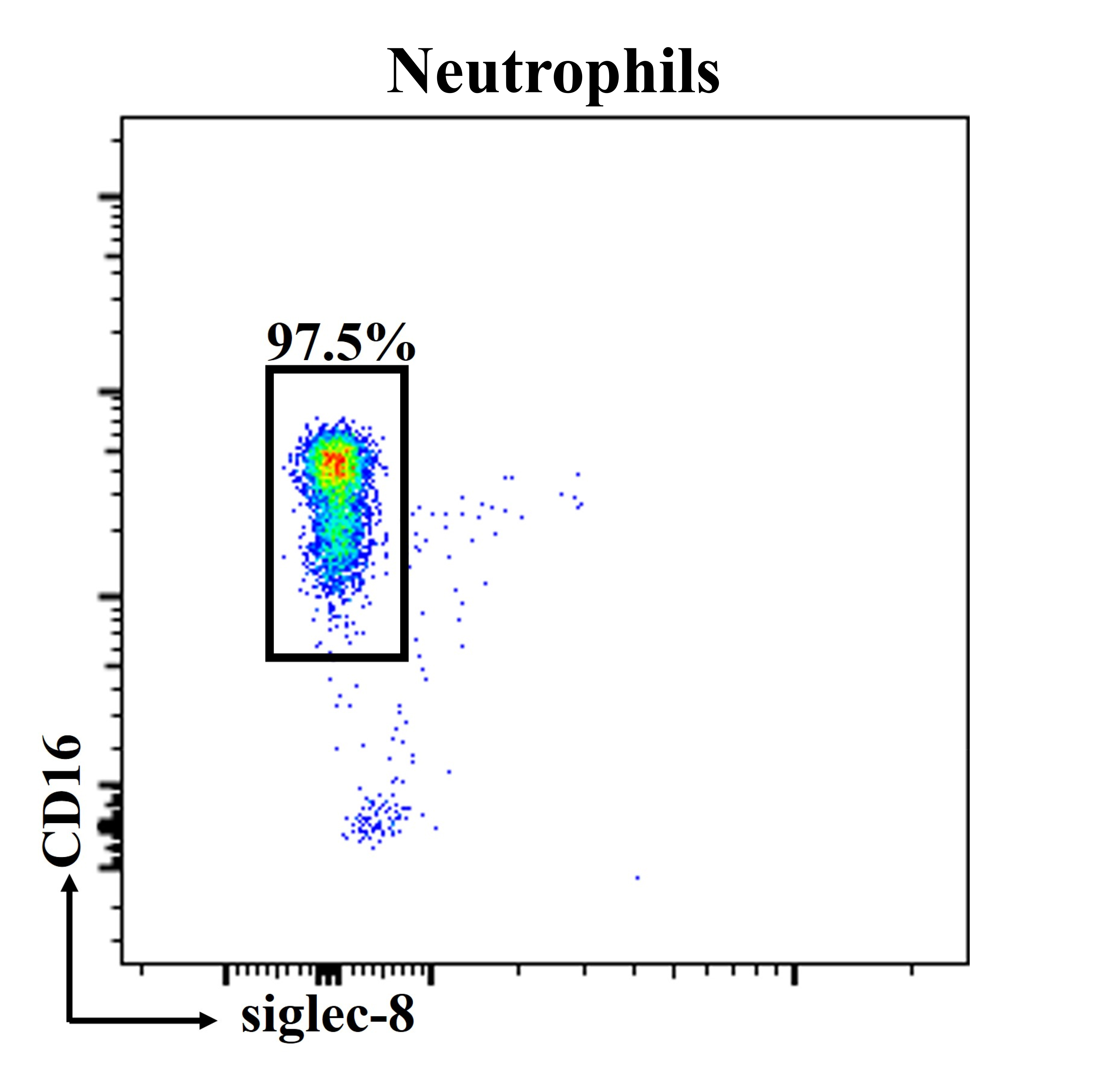

Supplement: Supplementary file 7 [file Image1.jpg]
