# Supplementary material for: Characterizing the Neutrophilic Inflammation in Chronic Rhinosinusitis With Nasal Polyps
Source: Front Cell Dev Biol. 2021 Dec 17;9:793073. doi: 10.3389/fcell.2021.793073 (PMC8718617; doi:10.3389/fcell.2021.793073)
Supplement: Supplementary file 8 [file DataSheet1.docx]

**Online Data Supplement**

**Characterizing the neutrophilic inflammation in chronic rhinosinusitis with nasal polyps**

**Running Head:** Neutrophilic inflammation in CRSwNP

Jian-Wen Ruan^1^, Jie-Fang Zhao^1^, Xue-Li Li^1^, Bo Liao^1^, Li Pan^1^, Ke-Zhang Zhu^1^, Qi-Miao Feng^1^, Jin-Xin Liu^1^, Zi-E Yu^1^, Jia Song^1^, Hai Wang^1*^, Zheng Liu^1*^

* Joint corresponding authors

^1^Department of Otolaryngology-Head and Neck Surgery, Tongji Hospital, Tongji Medical College, Huazhong University of Science and Technology, Wuhan, China

**Correspondence to:** Zheng Liu, M.D., Ph.D., Department of Otolaryngology-Head and Neck Surgery, Tongji Hospital, Tongji Medical College, Huazhong University of Science and Technology, No. 1095 Jiefang Avenue, Wuhan 430030, China. Tel: 86-27-83662606; Fax: 86-27-83662606; E-mail: [zhengliuent@hotmail.com](mailto:zhengliuent@hotmail.com);

or Hai Wang, M.D., Ph.D., Department of Otolaryngology-Head and Neck Surgery, Tongji Hospital, Tongji Medical College, Huazhong University of Science and Technology, No. 1095 Jiefang Avenue, Wuhan 430030, China. Tel: 86-13638601442; Fax: 86-27-83663681; E-mail: 814899096@qq.com

**MATERIALS AND METHODS**

**Subjects**

Eighty-two control subjects and 375 patients with chronic rhinosinusitis with nasal polyps (CRSwNP) were enrolled. CRSwNP was diagnosed according to the current guidelines (Fokkens et al., 2020; Orlandi et al., 2021). There were 32 control subjects and 249 CRSwNP patients involved in the clinical feature study, histology study of neutrophil distribution, and Bio-Plex suspension chip analysis of 34 inflammatory mediators. Immunofluorescence staining study of apoptotic neutrophils included 15 control subjects and 37 CRSwNP patients. Sixteen control subjects and 46 CRSwNP patients were involved in neutrophil culture with tissue homogenate study. Nineteen control subjects and 43 CRSwNP patients were involved in enzyme-linked immunosorbent assay (ELISA) of eosinophilic cationic protein (ECP) and myeloperoxidase (MPO). Detailed information regarding the demographics of these subjects is shown in Table E1.

The atopic status was assessed via skin prick test with a standard panel of inhalant allergens common in our region and/or the ImmunoCAP was used to detect IgE antibodies against common inhalant allergens (Phadia, Uppsala, Sweden) (Wang et al., 2016). Allergic rhinitis was diagnosed based on the concordance between typical history of allergic symptoms and atopy test results. Asthma was diagnosed by respiratory physicians according to the guidelines of the Global Initiative for Asthma (Bateman et al., 2008). Subjects with immunodeficiency, fungal sinusitis, cystic fibrosis, antrochoanal polyp, primary ciliary dyskinesia, systemic vasculitis, or acute upper respiratory tract infection within 1 month before enrollment, or those under immunotherapy were excluded. Given the low prevalence of aspirin-exacerbated respiratory disease in Chinese patients (Cao et al., 2009; Fan et al., 2012), we did not include these patients in this study. No patients received biologics. Oral and nasal glucocorticoids were discontinued for at least 3 months and 1 month before surgery, respectively.

For the 249 CRSwNP patients involved in the clinical and inflammatory feature study, baseline symptoms including nasal obstruction, rhinorrhea, headache, facial pain, loss of smell, and overall symptom burden were scored by the visual analog scale method before surgery, as previously described (Fokkens et al., 2020). The total symptom scores were calculated by summarizing the scores of the five individual symptom domain. Endoscopic physical findings including nasal polyps (NPs), edema, nasal discharge, scarring, and crusting were scored according to the Lund-Kennedy method (Fokkens et al., 2020). Computed tomography scans were graded based on the Lund-Mackay scoring system. After surgery, the patients were under recommended treatment and were followed up according to the current guidelines (Fokkens et al., 2020; Orlandi et al., 2021).

To validate the rationality of using inferior turbinate tissue as a control in neutrophil infiltration study, normal ethmoid sinus tissues were obtained from 30 subjects receiving surgery for sinus cyst, nasal tumour, or maxillofacial trauma and without rhinosinusitis or rhinitis. Among them, 15 subjects were recruited for immunoﬂuorescence staining experiment, 7 (47%) were male, 2 (13%) demonstrated atopy and none of the subjects had allergic rhinitis or asthma. The 25^th^ and 75^th^ percentile of age were 23.0 years and 57.0 years, respectively, with a median of 39.5 years. Fifteen subjects were involved in enzyme-linked immunosorbent assay (ELISA) experiment, 9 (60%) were male, 1 (7%) had atopy and none of the subjects had allergic rhinitis or asthma. The 25^th^ and 75^th^ percentile of age were 26.0 years and 49.5 years, respectively, with a median of 38.0 years.

In addition, 46 healthy subjects were recruited for peripheral blood neutrophil isolation and culture study (Table E2).

**Histology and** **immunoﬂuorescence staining**

Freshly obtained NP and control inferior turbinate tissues were fixed in 4% paraformaldehyde and embedded in paraffin. Paraffin-embedded sections (4 μm) were stained with hematoxylin and eosin to delineate the general pathological characteristics and infiltration of eosinophils, mononuclear cells, and plasma cells.

MPO-positive neutrophils and active caspase-3- and MPO-positive apoptotic neutrophils were studied using immunofluorescence staining. (Li et al., 2016) For immunofluorescence staining, after antigen retrieval and blocking, sections were stained with primary antibodies (Table E3) overnight at 4 °C and subsequent fluorescence-conjugated secondary antibodies for 40 minutes at room temperature in dark (Table E4). Species- and subtype-matched antibodies were used as negative controls. The number of eosinophils, mononuclear cells, plasma cells, MPO^+^ neutrophils, and active caspase-3^+^MPO^+^ apoptotic neutrophils in the lamina propria were counted at ×400 high-power magnification. Ten high-power fields were randomly selected and counted by two independent physicians, who were blind to the clinical data, as previously described (Cao et al., 2009; Wang et al., 2018). The immunofluorescence staining intensity of G-CSF in epithelium was analyzed by using image pro-plus 6.0 analysis software (Media Cybernetics, Inc. Silver Spring, MD, USA) (Jensen, 2013).

**Nasal tissue homogenate preparation**

For ELISA and Bio-Plex suspension chip analysis, tissue samples were weighed and 1 mL of 0.9% sodium chloride solution supplemented with 10 μL of 100 mM phenylmethylsulfonyl fluoride was added per 0.1 g tissue, as previously reported (Cao et al., 2014; Liao et al., 2018). For culture experiments, NP and control inferior turbinate mucosal tissues were weighed and every 0.1 g of tissue was placed in 0.1 mL of RPMI 1640 medium (Guge Biotechnology, Wuhan, China) with 1% penicillin/streptomycin (Guge Biotechnology) and 10% fetal calf serum (FCS) (Gibico, Thermo Fisher Scientific, Waltham, MA, USA), as previously described (Cao et al., 2014; Zhang et al., 2014). Next, the tissue was homogenized on ice and centrifuged at 3,000 rpm for 15 minutes at 4 °C. Supernatants were harvested and stored at −80 °C for future studies. The total protein levels in the tissue homogenates were measured using a BCA protein detection kit (Guge Biotechnology, Wuhan, China) (Cao et al., 2014).

**ELISA and Bio-Plex suspension chip**

MPO and ECP levels in the tissue homogenates were detected using commercial ELISA kits (Feiya Biological Technology, Jiangsu, China). The protein levels of interleukin (IL)-1β, IL-1 receptor antagonist (IL-1Ra), IL-2, IL-4, IL-5, IL-6, IL-7, IL-8, IL-9, IL-10, IL-12, IL-13, IL-15, IL-17A, IL-22, IL-25, IL-33, eotaxin, basic fibroblast growth factor, granulocyte colony-stimulating factor (G-CSF), interferon-γ (IFN-γ), IFN-γ-induced protein 10, monocyte chemoattractant protein-l, macrophage inflammatory protein (MIP)-1α, platelet-derived growth factor-BB, MIP-1β, tumor necrosis factor α, vascular endothelial growth factor, immunoglobulin (Ig) G1, IgG2, IgG3, IgG4, IgM, and IgE in supernatants were detected using the Bio-Plex suspension chip method (Bio-Rad, Hercules, Calif, USA) according to the manufacturer’s instructions (Liao et al., 2018). All the measured cytokine results were normalized to total tissue protein levels (Liao et al., 2015). Any values less than the detection limit were assigned a value equal to 1/10 of the detection limit (Liao et al., 2018). The total protein levels in tissue homogenates were measured using a BCA protein detection kit (Guge Biotechnology) (Cao et al., 2014).

**Neutrophil isolation and culture**

Neutrophils were purified from the peripheral blood of healthy subjects using Ficoll and 3% dextran (Tianjin Haoyang Biological manufacture, Tianjin, China), as mentioned previously (Calzetti et al., 2017; Wang et al., 2018). The peripheral blood was mixed with an equal volume of 3% dextran and phosphate-buffered saline (PBS) for 30 minutes. The leukocyte containing supernatants were then carefully layered onto Ficoll-Hypaque gradient (Tianjin Haoyang Biological manufacture). After centrifugation at 2000 rpm for 20 min, red cells and all the layers above the red cells were removed. Thereafter, the immunomagnetic negative selection step was performed using the ‘‘EasySep Human Neutrophil Enrichment Kit’’ (StemCell Technologies, Vancouver, British Columbia, Canada) (Wilkinson et al., 2018; Grunwell et al., 2019). The purity of the isolated CD16^+^Siglec-8^-^ neutrophils were consistently higher than 97%, as detected via flow cytometry (Fig E1).

For neutrophil culture experiment, 1×10^6^/well freshly isolated neutrophils were cultured in 100 μg tissue homogenate supernatants of control tissues, Neu-low NPs, and Neu-high NPs in 1 mL RPMI 1640 medium (Guge Biotechnology) with 1% penicillin/streptomycin (Guge Biotechnology) and 10% FCS (Gibico, Thermo Fisher Scientific, Waltham, MA, USA) in 12-well plates in a 5% CO_2_-humidified atmosphere at 37 °C for 8 h. In some experiments, purified neutrophils were cultured in homogenates from Neu-high NPs in the presence of anti-G-CSF (2 µg/mL, R&D Systems, Minneapolis, MN, USA), anti-IL-6 (2 µg/mL, R&D Systems), or IgG control (2 µg/mL, R&D Systems) (Table E6) (N Shirafuji1, 1989). Dexamethasone (10^-7^ M, Sigma-Aldrich, St. Louis, MO, USA) was used as a control and added to the culture with homogenates of control tissues.

**Flow cytometry**

Isolated peripheral blood neutrophils were stained with APC-conjugated mouse monoclonal antibody against human CD16 (5 µL/10^6^ cells; BioLegend, San Diego, CA, USA) and PE-conjugated mouse monoclonal antibody against human Siglec-8 (5 µL/10^6^ cells; BioLegend) for 30 minutes at 4 °C in dark according to the manufacturer's instructions to detect the purity. Cultured neutrophils were centrifuged at 2,000 rpm for 10 min, washed twice, and resuspended with 2% heat-inactivated fetal bovine serum/PBS. As previously described (Wilkinson et al., 2018), neutrophils were stained with fluorescein isothiocyanate-labelled anti-Annexin-V antibody (5 µL/10^6^ cells; BD Biosciences, San Jose, CA, USA) and 7-aminoactinomycin D (7-AAD; 5 µL/10^6^ cells; BD Biosciences) at 25 °C for 10 minutes according to the manufacturer’s instructions to study the apoptosis. The stained cells were analyzed using a BD LSR Fortessa™ X-20 flow cytometer (BD Biosciences). Data were analyzed using FlowJo software (TreeStar, Ashland, OR, USA).

**REFERENCES**

Bateman E.D., Hurd S.S., Barnes P.J., Bousquet J., Drazen J.M., FitzGerald J.M., et al. (2008). Global strategy for asthma management and prevention: GINA executive summary. *Eur Respir J* 31(1) 143-178. doi: 10.1183/09031936.00138707.

Calzetti F., Tamassia N., Arruda-Silva F., Gasperini S., Cassatella M.A. (2017). The importance of being "pure" neutrophils. *J Allergy Clin Immunol* 139(1) 352-355 e356. doi: 10.1016/j.jaci.2016.06.025.

Cao P.P., Li H.B., Wang B.F., Wang S.B., You X.J., Cui Y.H., et al. (2009). Distinct immunopathologic characteristics of various types of chronic rhinosinusitis in adult Chinese. *J Allergy Clin Immunol* 124(3) 478-484, 484 e471-472. doi: 10.1016/j.jaci.2009.05.017.

Cao P.P., Zhang Y.N., Liao B., Ma J., Wang B.F., Wang H., et al. (2014). Increased local IgE production induced by common aeroallergens and phenotypic alteration of mast cells in Chinese eosinophilic, but not non-eosinophilic, chronic rhinosinusitis with nasal polyps. *Clin Exp Allergy* 44(5) 690-700. doi: 10.1111/cea.12304.

Fan Y., Feng S., Xia W., Qu L., Li X., Chen S., et al. (2012). Aspirin-exacerbated respiratory disease in China: a cohort investigation and literature review. *Am J Rhinol Allergy* 26(1) e20-22. doi: 10.2500/ajra.2012.26.3738.

Fokkens W.J., Lund V.J., Hopkins C., Hellings P.W., Kern R., Reitsma S., et al. (2020). European Position Paper on Rhinosinusitis and Nasal Polyps 2020. *Rhinology* 58(Suppl S29) 1-464. doi: 10.4193/Rhin20.600.

Grunwell J.R., Stephenson S.T., Tirouvanziam R., Brown L.A.S., Brown M.R., Fitzpatrick A.M. (2019). Children with Neutrophil-Predominant Severe Asthma Have Proinflammatory Neutrophils With Enhanced Survival and Impaired Clearance. *J Allergy Clin Immunol Pract* 7(2) 516-525 e516. doi: 10.1016/j.jaip.2018.08.024.

Jensen E.C. (2013). Quantitative analysis of histological staining and fluorescence using ImageJ. *Anat Rec (Hoboken)* 296(3) 378-381. doi: 10.1002/ar.22641.

Li B., Zeng M., Zheng H., Huang C., He W., Lu G., et al. (2016). Effects of ghrelin on the apoptosis of human neutrophils in vitro. *Int J Mol Med* 38(3) 794-802. doi: 10.3892/ijmm.2016.2668.

Liao B., Cao P.P., Zeng M., Zhen Z., Wang H., Zhang Y.N., et al. (2015). Interaction of thymic stromal lymphopoietin, IL-33, and their receptors in epithelial cells in eosinophilic chronic rhinosinusitis with nasal polyps. *Allergy* 70(9) 1169-1180. doi: 10.1111/all.12667.

Liao B., Liu J.X., Li Z.Y., Zhen Z., Cao P.P., Yao Y., et al. (2018). Multidimensional endotypes of chronic rhinosinusitis and their association with treatment outcomes. *Allergy* 73(7) 1459-1469. doi: 10.1111/all.13411.

N Shirafuji1 S.A., S Matsuda KW, F Takaku, Nagata. (1989). A new bioassay for human granulocyte colony-stimulating factor (hG-CSF) using murine myeloblastic NFS-60 cells as targets and estimation of its levels in sera from normal healthy persons and patients with infectious and hematological disorders. *Exp Hematol.* 17 116-117.

Orlandi R.R., Kingdom T.T., Smith T.L., Bleier B., DeConde A., Luong A.U., et al. (2021). International consensus statement on allergy and rhinology: rhinosinusitis 2021. *Int Forum Allergy Rhinol* 11(3) 213-739. doi: 10.1002/alr.22741.

Wang H., Li Z.Y., Jiang W.X., Liao B., Zhai G.T., Wang N., et al. (2018). The activation and function of IL-36gamma in neutrophilic inflammation in chronic rhinosinusitis. *J Allergy Clin Immunol* 141(5) 1646-1658. doi: 10.1016/j.jaci.2017.12.972.

Wang Y., Chen H., Zhu R., Liu G., Huang N., Li W., et al. (2016). Allergic Rhinitis Control Test questionnaire-driven stepwise strategy to improve allergic rhinitis control: a prospective study. *Allergy* 71(11) 1612-1619. doi: 10.1111/all.12963.

Wilkinson A.N., Gartlan K.H., Kelly G., Samson L.D., Olver S.D., Avery J., et al. (2018). Granulocytes Are Unresponsive to IL-6 Due to an Absence of gp130. *J Immunol* 200(10) 3547-3555. doi: 10.4049/jimmunol.1701191.

Zhang N., Van Crombruggen K., Holtappels G., Lan F., Katotomichelakis M., Zhang L., et al. (2014). Suppression of cytokine release by fluticasone furoate vs. mometasone furoate in human nasal tissue ex-vivo. *PLoS One* 9(4) e93754. doi: 10.1371/journal.pone.0093754.

**Table E1. Demographic characteristics of the control subjects undergoing septoplasty and CRSwNP patients in different experiments**

|  | **Control subjects** | **Patients with Neu-high NPs** | **Patients with Neu-low NPs** | ***P* value** |
| --- | --- | --- | --- | --- |
|  | **(n = 82)** | **（n = 201）** | **(n = 174)** |  |
| **Methodology used**  ***Bio-Plex suspension chip, histology, and clinical feature study*** |  |  |  |  |
| Subject no. | 32 | 131 | 118 |  |
| Gender, male, N (%) | 19 (59%) | 90 (69%) | 76 (64%) | 0.554 |
| Age (years) | 41.0 (20.0–53.0) | 40.0 (25.0–51.0) | 44.0 (34.5–51.0) | 0.253 |
| Patients with atopy | 4 (13%) | 22 (17%) | 27 (23%) | 0.293 |
| Patients with AR | 0 (0%) | 16 (12%) | 15 (13%) | 0.075 |
| Patients with asthma | 0 (0%) | 9 (7%) | 18 (15%) | **0.010** |
| ***Immunofluorescence study*** |  |  |  |  |
| Subject no. | 15 | 19 | 18 |  |
| Gender, male, N (%) | 8 (53%) | 12 (63%) | 10 (56%) | 0.826 |
| Age (years) | 37.0 (24.0–52.0) | 38.0 (30.0–46.0) | 43.0 (36.0–50.8) | 0.091 |
| Patients with atopy | 2 (13%) | 4 (21%) | 3 (17%) | 0.902 |
| Patients with AR | 0 (0%) | 1 (5%) | 2 (11%) | 0.629 |
| Patients with asthma | 0 (0%) | 1 (5%) | 2 (11%) | 0.629 |
| ***In vitro* experiment** |  |  |  |  |
| Subject no. | 16 | 30 | 16 |  |
| Gender, male, N (%) | 9（56%） | 18（60%） | 8 (50%) | 0.809 |
| Age (years) | 40.5 (21.0–54.3） | 41.5 (34.0–50.0) | 40.5 (36.0–48.0) | 0.570 |
| Patients with atopy | 1 (6%) | 6 (20%) | 2 (13%) | 0.529 |
| Patients with AR | 0 (0%) | 1 (3%) | 1 (6%) | 1.000 |
| Patients with asthma | 0 (0%) | 1 (3%) | 2 (13%) | 0.322 |
| ***ELISA*** |  |  |  |  |
| Subject no. | 19 | 21 | 22 |  |
| Gender, male, N (%) | 10 (53%) | 14 (67%) | 13 (59%) | 0.663 |
| Age (years) | 39.0 (23.0–53.0) | 41.0 (27.0–55.0) | 38.5 (28.0–51.5) | 0.755 |
| Patients with atopy | 1 (5%) | 4 (19%) | 5 (23%) | 0.315 |
| Patients with AR | 0 (0%) | 1 (5%) | 3 (14%) | 0.314 |
| Patients with asthma | 0 (0%) | 1 (5%) | 2 (9%) | 0.768 |

NP, nasal polyp; Neu-high, neutrophil-high; Neu-low, neutrophil-low; AR, allergic rhinitis; no, number. For continuous variables, results are expressed as medians and interquartile ranges. Categorical variables are summarized using frequency and percentage. The *P* values in bold indicate the values less than 0.05.

**Table E2. Demographics of the healthy subjects involved in peripheral blood neutrophil isolation and culture study**

|  | **Healthy subjects** |
| --- | --- |
| Subject no. | 46 |
| Gender, male, N (%) | 26 (57%) |
| Age (years) | 33.0 (25.0–46.3) |
| Patients with atopy | 5 (11%) |
| Patients with AR | 0 (0%) |
| Patients with asthma | 0 (0%) |

AR, allergic rhinitis; no, number. For continuous variables, results are expressed as medians and interquartile ranges. Categorical variables are summarized using frequency and percentage.

**Table E3. Primary antibodies used in immunofluorescence staining**

| **Antibody** | **Species** | **Concentration** | **Clone ID** | **Reference** | **Source** |
| --- | --- | --- | --- | --- | --- |
| MPO | Goat | 1:200 | Polyclonal | AF3667 | R&D (Minneapolis, MN, USA) |
| Active caspase- 3 | Rabbit | 1:100 | Polyclonal | ab2302 | Abcam (Cambridge, MA, UK) |
| G-CSF | Rabbit | 1:100 | EPR3203(N)(B) | ab181053 | Abcam |
| IgG | Goat | 1:100 | Polyclonal | AB-108-C | R&D |
| IgG | Rabbit | 1:100 | Polyclonal | AB-105-C | R&D |

MPO, myeloperoxidase; G-CSF, granulocyte colony-stimulating factor; Ig, immunoglobulin.

**Table E4. Secondary antibodies used in immunofluorescence staining**

| **Antibody** | **Concentration** | **Clone** | **Reference** | **Source** |
| --- | --- | --- | --- | --- |
| IFKine™ Green donkey anti-goat IgG | 1:100 | polyclonal | A24231 | Abbkine Scientific Company (Wuhan, China) |
| IFKine™ Red donkey anti-rabbit IgG | 1:100 | polyclonal | A24421 | Abbkine |

**Table E5. Detection limits for ELISA and Bio-Plex suspension chip assay**

| **Target** | **Detection limit** | **Target** | **Detection limit** |
| --- | --- | --- | --- |
| IL-1β | 0.6 pg/mL | bFGF | 1.9 pg/mL |
| IL-1Ra | 5.5 pg/mL | G-CSF | 1.7 pg/mL |
| IL-2 | 1.6 pg/mL | IFN-γ | 6.4 pg/mL |
| IL-4 | 0.7 pg/mL | IP-10 | 6.1 pg/mL |
| IL-5 | 0.6 pg/mL | MCP-1 | 1.1 pg/mL |
| IL-6 | 2.6 pg/mL | MIP-1α | 1.6 pg/mL |
| IL-7 | 1.1 pg/mL | PDGF-BB | 2.9 pg/mL |
| IL-8 | 1.0 pg/mL | MIP-1β | 2.4 pg/mL |
| IL-9 | 2.5 pg/mL | TNF-α | 6.0 pg/mL |
| IL-10 | 0.3 pg/mL | VEGF | 3.1 pg/mL |
| IL-12 | 3.5 pg/mL | IgG1 | 0.0264 ng/mL |
| IL-13 | 0.7 pg/mL | IgG2 | 0.6615 ng/mL |
| IL-15 | 2.4 pg/mL | IgG3 | 0.0030 ng/mL |
| IL-17A | 3.3 pg/mL | IgG4 | 0.0021 ng/mL |
| IL-22 | 0.9 pg/mL | IgM | 0.1473 ng/mL |
| IL-25 | 0.8 pg/mL | IgE | 0.0040 ng/mL |
| IL-33 | 1.6 pg/mL | MPO | 2.0 pg/mL |
| Eotaxin | 2.5 pg/mL | ECP | 80.0 pg/mL |

bFGF, basic fibroblast growth factor; G-CSF, granulocyte colony-stimulating factor; IFN-γ, interferon γ; Ig, immunoglobulin; IL, interleukin; IL-1Ra, IL-1 receptor antagonist; IP-10, interferon-γ-induced protein-10; MCP-1, monocyte chemoattractant protein-1; MIP, macrophage inflammatory protein; PDGF-BB, platelet-derived growth factor-BB; TNF-α, tumor necrosis factor α; VEGF, vascular endothelial growth factor.

**Table E6. Neutralizing antibodies in cell experiments**

| **Antibody** | **Species** | **Concentration** | **Clone ID** | **Reference** | **Source** |
| --- | --- | --- | --- | --- | --- |
| G-CSF | Goat | 2 μg/mL | polyclonal | AF-214-NA | R&D systems  (Minneapolis, MN, USA) |
| IL-6 | Goat | 2 μg/mL | polyclonal | AF-206-NA | R&D systems |
| IgG | Goat | 2 μg/mL | polyclonal | AB-108-C | R&D systems |

G-CSF, granulocyte colony-stimulating factor; Ig, immunoglobulin; IL, interleukin.

|  | **Control tissue**  **(n = 32)** | **Neu-high NP**  **(n = 131)** | **Neu-low NP**  **(n = 118)** | **Control tissue vs**  **Neu-high NP**  ***P* value** | **Control tissue vs**  **Neu-low NP**  ***P* value** | **Neu-high NP vs**  **Neu-low NP**  ***P* value** |
| --- | --- | --- | --- | --- | --- | --- |
| Eosinophil /HPF | 2.00  (1.00–3.25) | 2.50  (0.75–19.15) | 13.00  (2.50–59.75) | 0.438 | **<0.001** | <**0.001** |
| Plasma cell /HPF | 1.00  (1.50–3.00) | 5.00  (2.00–8.00) | 6.50  (3.00–7.00) | **<0.001** | **<0.001** | 0.743 |
| Mononuclear cell /HPF | 18.50  (11.00–36.25) | 41.00  (23.00–61.00) | 42.50  (22.00–73.00) | **<0.001** | **<0.001** | 0.392 |

**Table E7. Histological study of Neu-high and Neu-low NPs**

NP, nasal polyp; Neu-high, neutrophil-high; Neu-low, neutrophil-low; HPF, high-power field. The *P* values in bold indicate the values less than 0.05.

**Table E8. Tissue levels of inflammatory mediators detected using the Bio-Plex assay**

|  | **Control tissue**  **(n = 32)** | **Neu-high NP**  **(n = 131)** | **Neu-low NP**  **(n = 118)** | **Control tissue vs**  **Neu-high NP**  ***P* value** | **Control tissue vs Neu-low NP**  ***P* value** | **Neu-high NP vs Neu-low NP**  ***P* value** |
| --- | --- | --- | --- | --- | --- | --- |
| IL-1β (pg/mg) | 8.04  (4.74–12.78) | 15.55  (9.41–31.53) | 10.61  (5.00–19.37) | **< 0.001** | 0.265 | **< 0.001** |
| IL-1Ra (ng/mg) | 1.03  (0.57–1.67) | 1.92  (1.21–3.16) | 1.49  (0.85–2.43) | **< 0.001** | 0.058 | **0.027** |
| IL-2 (pg/mg) | 2.25  (1.61–2.64) | 1.50  (1.09–2.47) | 1.61  (0.93–2.38) | 0.064 | 0.069 | 0.632 |
| IL-4 (pg/mg) | 85.94  (51.54–106.39) | 109.81  (79.54–151.32) | 110.92  (71.34–180.13) | **0.003** | **0.008** | 1.000 |
| IL-5 (pg/mg) | 18.49  (11.20–26.02) | 13.58  (8.58–35.40) | 47.41  (13.99–129.75) | 1.000 | **<0.001** | **0.014** |
| IL-6 (pg/mg) | 25.57  (8.25–184.95) | 160.88  (66.66–279.22) | 60.38  (27.63–137.50) | **< 0.001** | 0.534 | **<0.001** |
| IL-7 (pg/mg) | 207.04  (157.44–289.58) | 277.17  (193.86–438.19) | 334.07  (191.56–512.72) | **0.043** | **0.011** | 1.000 |
| IL-8 (pg/mg) | 218.51  (89.01–392.93) | 1253.11  (628.17–1814.93) | 338.04  (226.29–641.01) | **< 0.001** | 0.052 | **< 0.001** |
| IL-9 (pg/mg) | 53.29  (37.69–77.56) | 100.33  (66.66–192.32) | 113.27  (59.08–515.50) | **< 0.001** | **< 0.001** | 1.000 |
| IL-10 (pg/mg) | 68.17  (30.42–344.30) | 72.04  (21.86–113.86) | 62.70  (6.19–151.04) | 0.395 | 0.888 | 1.000 |
| IL-12 (pg/mg) | 17.76  (4.49–71.84) | 51.67  (30.55–77.91) | 56.15  (30.11–104.59) | 0.231 | 0.190 | 1.000 |
| IL-13 (pg/mg) | 32.16  (23.24–51.18) | 36.01  (21.65–65.62) | 63.00  (25.81–210.63) | 1.000 | **0.029** | **0.016** |
| IL-15 (pg/mg) | 13.14  (10.91–20.50) | 12.54  (9.05–18.34) | 11.75  (7.40–19.40) | 1.000 | 0.408 | 0.962 |
| IL-17A (pg/mg) | 168.34  (120.97–270.68) | 239.13  (135.91–342.19) | 240.08  (178.33–331.48) | **0.036** | **0.002** | 0.575 |
| IL-22 (pg/mg) | 10.65  (7.34–23.05) | 8.01  (4.82–13.62) | 8.63  (5.66–18.35) | 0.129 | 0.738 | 0.560 |
| IL-25 (pg/mg) | 1.64  (0.71–3.42) | 2.16  (1.36–3.52) | 2.00  (1.43–3.92) | 0.275 | 0.128 | 1.000 |
| IL-33 (ng/mg) | 11.12  (4.91–14.25) | 6.12  (3.11–9.33) | 5.28  (3.17–9.19) | 0.210 | 0.088 | 1.000 |
| Eotaxin (pg/mg) | 291.12  (150.84–963.72) | 276.41  (170.36–517.78) | 261.02  (161.30–723.75) | 1.000 | 1.000 | 1.000 |
| bFGF (ng/mg) | 1.23  (0.74–1.93) | 0.99  (0.57–1.59) | 0.84  (0.58–1.31) | 0.653 | 0.075 | 0.328 |
| G-CSF (pg/mg) | 164.44  (57.12–462.01) | 1141.11  (328.99–3217.19) | 187.86  (80.14–507.60) | **< 0.001** | 1.000 | **< 0.001** |
| IFN-γ (pg/mg) | 182.94  (151.53–267.10) | 261.57  (156.66–364.01) | 277.69  (178.44–324.61) | **0.022** | **0.031** | 1.000 |
| IP-10 (ng/mg) | 3.91  (1.96–10.09) | 2.74  (1.35–5.61) | 2.49  (1.27–4.27) | 0.540 | 0.136 | 0.867 |
| MCP-1 (pg/mg) | 408.55  (249.57–647.73) | 632.06  (500.26–952.99) | 502.37  (351.14–1038.99) | **0.002** | 0.225 | **0.031** |
| MIP-1α (pg/mg) | 12.4  (9.65–17.92） | 25.25  (14.25–45.80) | 18.58  (8.58–31.44) | **< 0.001** | 0.057 | **0.013** |
| PDGF-BB (pg/mg) | 260.64  (139.75–537.63) | 177.89  (139.17–193.65) | 207.21  (105.01–365.07) | 0.129 | 0.116 | 1.000 |
| MIP-1β (pg/mg) | 178.99  (114.17–391.44) | 432.33  (292.41–787.34) | 419.36  (245.47–696.84) | **< 0.001** | **< 0.001** | 0.909 |
| TNF-α (pg/mg) | 176.99  (112.78–274.02) | 141.82  (100.76–219.96) | 128.53  (85.24–208.17) | 0.707 | 0.181 | 0.806 |
| VEGF (ng/mg) | 0.22  (0.06–1.44) | 1.15  (0.73–2.02) | 1.14  (0.47–2.21) | **< 0.001** | **0.001** | 1.000 |
| IgG1 (μg/mg) | 35.52  (23.08–61.55) | 58.26  (42.90–83.74) | 49.47  (36.00–71.77) | **< 0.001** | **< 0.001** | 0.586 |
| IgG2 (μg/mg) | 16.37  (14.38–30.64) | 29.48  (20.42–46.00) | 30.59  (21.71–55.02) | **0.003** | **0.005** | 1.000 |
| IgG3 (μg/mg) | 21.62  (13.54–33.38) | 19.21  (13.05–32.63) | 14.34  (7.158–28.99) | 1.000 | 0.104 | 0.053 |
| IgG4 (μg/mg) | 2.97  (1.67–6.06) | 4.53  (2.36–9.06) | 4.48  (2.05–8.35) | 0.071 | 0.119 | 1.000 |
| IgM (μg/mg) | 5.89  (3.90–9.88) | 5.48  (3.89–9.26) | 5.83  (2.79–8.27) | 1.000 | 1.000 | 1.000 |
| IgE (ng/mg) | 6.14  (1.25–25.33) | 15.17  (4.92–40.55) | 30.24  (7.89–66.75) | 0.264 | **0.003** | **0.032** |

NP, nasal polyp; Neu-high, neutrophil-high; Neu-low, neutrophil-low; bFGF, basic fibroblast growth factor; G-CSF, granulocyte colony-stimulating factor; IFN-γ, interferon-γ; Ig, immunoglobulin; IL, interleukin; IL-1Ra, IL-1 receptor antagonist; IP-10, interferon-γ-induced protein-10; MCP-1, monocyte chemoattractant protein-1; MIP, macrophage inflammatory protein; PDGF-BB, platelet-derived growth factor-BB; TNF-α, tumor necrosis factor α; VEGF, vascular endothelial growth factor. Results are expressed as medians and interquartile ranges. The *P* values in bold indicate the values less than 0.05.

**Table E9. Univariate logistic analysis of factors associated with difficult-to-treat NPs**

|  | **Difficult-to-treat NPs** | **Non-difficult-to-treat NPs** | **OR (95% CI)** | ***P* value** |
| --- | --- | --- | --- | --- |
|  | **(n = 83)** | **(n = 166)** |  |  |
| Gender, male, N (%) | 47 (57%) | 119 (72%) | 0.516  (0.298–0.894) | **0.018** |
| Age | 39.00 (25.00–48.50) | 44.00 (27.00–52.75) | 0.980  (0.963–0.997) | **0.024** |
| Patients with atopy | 25 (30%) | 53 (32%) | 1.088  (0.615–1.927) | 0.204 |
| Patients with AR | 13 (16%) | 18 (11%) | 1.527  (0.708–3.291) | 0.280 |
| Patients with asthma | 18 (22%) | 6 (4%) | 7.385  (2.805–19.439) | **< 0.001** |
| Disease duration | 5.00 (2.00–10.00) | 4.00 (1.00–9.75) | 1.009  (0.976–1.042) | 0.613 |
| Patients with prior surgery | 47 (57%) | 42 (25%) | 3.854  (2.207–6.732) | **< 0.001** |
| Nasal obstruction score | 8.00 (6.00–9.50) | 6.00 (4.00–8.00) | 1.212  (1.089–1.349) | **< 0.001** |
| Headache score | 3.00 (0.00–5.50) | 2.00 (0.00–5.00) | 1.121  (1.023–1.229) | **0.015** |
| Facial pain score | 0.00 (0.00–3.00) | 0.00 (0.00–1.75) | 1.093  (0.981–1.217) | 0.106 |
| Loss of smell score | 8.00 (5.00–10.00) | 5.00 (1.00–8.75) | 1.156  (1.070–1.248) | **< 0.001** |
| Rhinorrhea score | 7.00 (3.00–8.00) | 5.00 (2.00–7.00) | 1.114  (1.020–1.215) | **0.016** |
| Total symptom score | 28.00 (18.00–31.00) | 18.00 (12.25–25.00) | 1.079  (1.045–1.115) | **< 0.001** |
| Overall burden score | 7.00 (5.00–8.00) | 6.00 (5.00–8.00) | 1.173  (1.033–1.333) | **0.014** |
| Nasal polyp score | 4.00 (2.00–4.50) | 4.00 (2.00–4.00) | 1.270  (1.054–1.529) | **0.012** |
| Bilateral CT score | 22.00 (19.00–24.00) | 14.00 (9.00–18.75) | 1.196  (1.131–1.264) | **< 0.001** |
| Total endoscopic score | 10.00 (8.00–12.00) | 8.00 (6.00–10.00) | 1.288 (1.166–1.423) | **< 0.001** |
| Blood leukocyte count (×10^9^/L) | 6.20 (5.47–7.48) | 6.14 (5.19–7.08) | 1.138  (0.979–1.322) | 0.091 |
| Blood neutrophil count (×10^9^/L) | 3.37 (2.81–4.09) | 3.22 (2.61–3.82) | 1.255  (1.051–1.499) | **0.012** |
| Blood neutrophil percent (%) | 53.70 (47.05–60.60) | 53.30 (48.03-58.00) | 1.006  (0.976–1.036) | 0.706 |
| Blood lymphocyte count (×10^9^/L) | 2.12 (1.80–2.70) | 2.03 (1.71–2.39) | 1.540  (1.028–2.307) | **0.036** |
| Blood lymphocyte percent (%) | 33.50 (28.35–41.90) | 34.20 (28.93–39.78) | 1.005  (0.976–1.035) | 0.742 |
| Blood eosinophil count (×10^9^/L) | 0.26 (0.12–0.58) | 0.21 (0.12–0.36) | 2.884  (1.240–6.706) | **0.014** |
| Blood eosinophil percent (%) | 3.80 (1.90–9.15) | 3.40 (1.70–5.38) | 1.076  (1.012–1.144) | **0.019** |
| Blood monocyte count (×10^9^/L) | 0.46 (0.37–0.62) | 0.48 (0.37–0.60) | 1.463  (0.749–2.858) | 0.266 |
| Blood monocyte percent (%) | 7.40 (6.20–9.15) | 7.60 (6.40–9.10) | 1.030  (0.986–1.076) | 0.185 |
| Tissue eosinophil number/HPF | 10.00 (2.50–38.75) | 2.75 (1.00–51.25) | 1.005  (1.000–1.009) | **0.032** |
| Tissue neutrophil number/HPF | 11.00 (6.16–28.21) | 11.00 (6.00–20.91) | 1.026  (1.007–1.045) | **0.007** |
| IL-1β (pg/mg) | 11.74 (5.64–34.34) | 13.63 (6.54–23.65) | 1.003  (0.995–1.012) | 0.441 |
| IL-1Ra (ng/mg) | 1.72 (0.87–2.93) | 1.60 (1.12–2.88) | 1.000  (1.000–1.000) | 0.603 |
| IL-2 (pg/mg) | 1.61 (1.08–2.45) | 1.51 (1.00–2.38) | 0.998  (0.884–1.104) | 0.832 |
| IL-4 (pg/mg) | 115.35 (78.12–180.76) | 107.59 (73.04–153.44) | 1.001  (0.999–1.002) | 0.322 |
| IL-5 (pg/mg) | 25.27 (10.73–91.20) | 15.55 (8.92–54.07) | 1.002  (1.001–1.004) | **0.008** |
| IL-6 (pg/mg) | 113.35 (46.31–228.79) | 104.31 (51.55–227.03) | 1.000  (0.999–1.000) | 0.355 |
| IL-7 (pg/mg) | 307.17 (195.62–494.20) | 283.72 (189.09–465.69) | 1.000  (1.000–1.001) | 0.209 |
| IL-8 (pg/mg) | 741.68 (305.68–1856.14) | 639.42 (328.78–1326.66) | 1.000  (1.000–1.000) | 0.386 |
| IL-9 (pg/mg) | 113.05 (65.65–212.73) | 100.95 (65.69–276.34) | 1.000  (1.000–1.000) | 0.581 |
| IL-10 (pg/mg) | 59.07 (3.44–123.58) | 71.92 (19.42–122.99) | 1.000  (0.999–1.001) | 0.740 |
| IL-12 (pg/mg) | 60.27 (30.87–95.98) | 52.14 (27.15–80.21) | 1.000  (0.997–1.003) | 0.886 |
| IL-13 (pg/mg) | 55.80 (29.12–129.41) | 32.73 (21.60–123.03) | 1.001  (1.000–1.001) | **0.036** |
| IL-15 (pg/mg) | 12.58 (8.91–19.08) | 11.88 (8.04–18.87) | 0.998  (0.979–1.017) | 0.835 |
| IL-17A (pg/mg) | 246.57 (181.01–415.73) | 235.00 (166.22–340.64) | 1.000  (0.999–1.001) | 0.892 |
| IL-22 (pg/mg) | 8.18 (4.80–13.54) | 8.46 (5.61–16.63) | 0.987  (0.968–1.006) | 0.186 |
| IL-25 (pg/mg) | 2.17 (1.23–4.15) | 2.08 (1.52–3.71) | 1.002  (0.997–1.007) | 0.407 |
| IL-33 (ng/mg) | 5.91 (3.12–9.80) | 5.57 (3.17–8.60) | 1.000  (1.000–1.000) | 0.219 |
| Eotaxin (pg/mg) | 302.57 (168.35–549.66) | 252.06 (166.31–590.30) | 1.000  (1.000–1.000) | 0.493 |
| bFGF (ng/mg) | 0.83 (0.54–1.29) | 0.92 (0.63–1.64) | 1.000  (1.000–1.000) | 0.961 |
| G-CSF (pg/mg) | 674.21 (179.20–2276.78) | 421.69 (113.46–1578.36) | 1.000  (1.000–1.000) | 0.137 |
| IFN-γ (pg/mg) | 278.02 (183.38–408.34) | 270.29 (160.59–344.08) | 1.000  (1.000–1.001) | 0.343 |
| IP-10 (ng/mg) | 2.58 (1.17–3.60) | 2.72 (1.41–5.76) | 1.000  (1.000–1.000) | 0.088 |
| MCP-1 (pg/mg) | 625.80 (421.60–1058.71) | 587.50 (396.05–1001.89) | 1.000  (1.000–1.001) | 0.620 |
| MIP-1α (pg/mg) | 24.54 (9.66–46.22) | 21.47 (12.24–37.17) | 0.995  (1.000–1.006) | 0.932 |
| PDGF-BB (pg/mg) | 215.18 (109.40–360.21) | 183.35 (139.92–318.20) | 1.000  (1.000–1.001) | 0.514 |
| MIP-1β (pg/mg) | 527.24 (250.23–885.22) | 395.49 (263.81–651.51) | 1.000  (0.999–1.001) | 0.520 |
| TNF-α (pg/mg) | 131.98 (100.79–253.70) | 139.17 (95.77–208.63) | 1.001  (0.999–1.002) | 0.664 |
| VEGF (ng/mg) | 1.37 (0.63–2.04) | 1.08 (0.59–2.12) | 1.000  (1.000–1.000) | 0.662 |
| IgG1 (μg/mg) | 57.69 (36.15–81.57) | 55.54 (41.90–76.51) | 1.000  (0.996–1.004) | 0.915 |
| IgG2 (μg/mg) | 31.35 (19.70–49.92) | 30.33 (21.33–45.96) | 1.001  (0.995–1.007) | 0.884 |
| IgG3 (μg/mg) | 17.08 (9.50–33.67) | 17.67 (9.92–28.76) | 1.006  (0.995–1.016) | 0.316 |
| IgG4 (μg /mg) | 6.43 (2.27–11.78) | 4.33 (2.13–6.90) | 1.040  (1.012–1.068) | **0.005** |
| IgM (μg/mg) | 6.68 (4.84–10.12) | 5.43 (3.22–7.99) | 1.074  (0.995–1.159) | 0.067 |
| IgE (ng/mg) | 26.93 (8.05–73.49) | 16.83 (5.45–40.73) | 1.007  (0.990–1.024) | 0.990 |

NP, nasal polyp; Neu-high, neutrophil-high; Neu-low, neutrophil-low; AR, allergic rhinitis; CT, computed tomography; bFGF, basic fibroblast growth factor; G-CSF, granulocyte colony-stimulating factor; IFN-γ, interferon-γ; Ig, immunoglobulin; IL, interleukin; IL-1Ra, IL-1 receptor antagonist; IP-10, interferon-γ-induced protein-10; MCP-1, monocyte chemoattractant protein-1; MIP, macrophage inflammatory protein; PDGF-BB, platelet-derived growth factor-BB; TNF-α, tumor necrosis factor α; VEGF, vascular endothelial growth factor; HPF, high-power field. For continuous variables, results are expressed as medians and interquartile ranges. Categorical variables are summarized using frequency and percentage. The *P* values in bold indicate the values less than 0.05.

**FIGURE LEGENDS**

**Fig E1. Representative flow plot shows the** **purity of isolated blood neutrophils.** Neutrophils are defined as CD16^+^Siglec-8^-^ cells.

**Fig E2. Comparison of neutrophil infiltration in inferior turbinate and ethmoid sinus tissues. A,** Tissue neutrophil numbers in inferior turbinate and ethmoid tissue mucosal tissue. Original magnification × 400. Scale bar, 100 μm. Arrows denote representative positive cells. **B,** Protein levels of MPO in inferior turbinate and ethmoid tissue mucosal tissues as detected by ELISA. IT, inferior turbinate; ET, ethmoid tissue; MPO, myeloperoxidase; HPF, high-power field.

**Fig E3.** **Protein levels of MPO (A) and ECP (B) in control nasal tissues, Neu-low NPs, and Neu-high NPs,** **as detected via ELISA.** NP, nasal polyp; Neu-high, neutrophil-high; Neu-low, neutrophil-low; ECP, eosinophilic cationic protein; MPO, myeloperoxidase.

**Fig E4.** **Representative photomicrographs show immunoﬂuorescence staining with isotype controls for primary anti-active caspase 3 and anti-MPO antibodies.** Original magnification ×400. Scale bar, 100 μm. MPO, myeloperoxidase.

**Fig E5.** **Dexamethasone inhibits the apoptosis of neutrophils. A,** Blood neutrophils (1 × 10^6^/well) from healthy subjects were cultured in control tissue homogenates in the presence or absence of dexamethasone (10^-7^ M) for 8 h; then, the apoptosis of neutrophils was analyzed via flow cytometry. Representative flow cytometric analysis of Annexin-V^+^7-AAD^-^ apoptotic, Annexin-V^-^7-AAD^-^ live, and Annexin-V^+^7-AAD^+^ necrotic neutrophils are shown. **B,** The mean frequencies of apoptotic, live, and necrotic neutrophils in different experimental groups are shown. **C-E**, The frequencies of Annexin-V^+^7-AAD^-^ apoptotic neutrophils **(C)**, Annexin-V^-^7-AAD^-^ live neutrophils **(D)**, and Annexin-V^+^7-AAD^+^ necrotic neutrophils **(E)** after culturing in the medium control and the homogenates of control tissues in the presence or absence of dexamethasone. The data are analyzed in a paired fashion. DEX, dexamethasone.

**Fig E6. The expression of G-CSF in sinonasal tissues. A,** Representative photomicrographs show G-CSF immunoreactivity in sinonasal tissues. Original magnification ×400. Scale bar, 100 μm. **B,** Quantification of immunofluorescence staining [intensity](javascript:;) of G-CSF in epithelium. NP, nasal polyp; Neu-high, neutrophil-high; Neu-low, neutrophil-low; G-CSF, granulocyte colony-stimulating factor.

**Fig E7. The correlation between tissue IL-6 and G-CSF levels in total CRSwNP patients.** IL, interleukin; G-CSF, granulocyte colony-stimulating factor; CRSwNP, chronic rhinosinusitis with nasal polyps.
